# Computer-Aided Design, Synthesis, and Antiviral Evaluation of Novel Acrylamides as Potential Inhibitors of E3-E2-E1 Glycoproteins Complex from Chikungunya Virus

**DOI:** 10.3390/ph13070141

**Published:** 2020-06-30

**Authors:** Gabriel Felipe Silva Passos, Matheus Gabriel Moura Gomes, Thiago Mendonça de Aquino, João Xavier de Araújo-Júnior, Stephannie Janaina Maia de Souza, João Pedro Monteiro Cavalcante, Elane Conceição dos Santos, Ênio José Bassi, Edeildo Ferreira da Silva-Júnior

**Affiliations:** 1 Laboratory of Medicinal Chemistry, Pharmaceutical Sciences Institute, Federal University of Alagoas, Maceió 57072-970, Brazil; gabrielfelipepassos@gmail.com (G.F.S.P.); matheus_gabriel199@hotmail.com (M.G.M.G.); jotaaraujo2004@gmail.com (J.X.d.A.-J.); 2 Center of Analysis and Research in Nuclear Magnetic Resonance, Chemistry and Biotechnology Institute, Federal University of Alagoas, Maceió 57072-970, Brazil; thiago.aquino@iqb.ufal.br; 3 Immunoregulation Research Group, Laboratory of Research in Virology and Immunology, Institute of Biological and Health Sciences, Federal University of Alagoas, Maceió 57072-970, Brazil; stephanniemaia92@gmail.com (S.J.M.d.S.); j.p.monteirocavalcante@gmail.com (J.P.M.C.); elane.santos@icbs.ufal.br (E.C.d.S.); enio.bassi@icbs.ufal.br (Ê.J.B.)

**Keywords:** virtual screening, acrylamides, chikungunya virus, antiviral, molecular docking, E3-E2-E1 glycoproteins complex

## Abstract

Chikungunya virus (CHIKV) causes an infectious disease characterized by inflammation and pain of the musculoskeletal tissues accompanied by swelling in the joints and cartilage damage. Currently, there are no licensed vaccines or chemotherapeutic agents to prevent or treat CHIKV infections. In this context, our research aimed to explore the potential in vitro anti-CHIKV activity of acrylamide derivatives. In silico methods were applied to 132 Michael’s acceptors toward the six most important biological targets from CHIKV. Subsequently, the ten most promising acrylamides were selected and synthesized. From the cytotoxicity MTT assay, we verified that LQM330, 334, and 336 demonstrate high cell viability at 40 µM. Moreover, these derivatives exhibited anti-CHIKV activities, highlighting the compound LQM334 which exhibited an inhibition value of 81%. Thus, docking simulations were performed to suggest a potential CHIKV-target for LQM334. It was observed that the LQM334 has a high affinity towards the E3-E2-E1 glycoproteins complex. Moreover, LQM334 reduced the percentage of CHIKV-positive cells from 74.07 to 0.88%, 48h post-treatment on intracellular flow cytometry staining. In conclusion, all virtual simulations corroborated with experimental results, and LQM334 could be used as a promising anti-CHIKV scaffold for designing new drugs in the future.

## 1. Introduction

The Chikungunya virus (CHIKV) is an arbovirus from the *Alphavirus* genus, which belongs to the *Togaviridae* family [1,2]. It is mainly transmitted by the bite of infected mosquitoes from *Aedes aegypti* and *Ae. albopictus* species [3,4,5,6,7,8]; although, *Ae. furcifer* and *Culex* spp. mosquitoes have been also reported as vectors [3,9,10,11]. Since 1990, CHIKV infections have been reported in many countries from South and Central Americas, estimating 11,675 million cases [2,12,13,14]. Recently, it was verified that the CHIKV is responsible for infecting people in more than 60 countries [15,16]. CHIKV is rarely fatal, whereas from 87 to 95% of infected patients are affected with debilitating arthralgia, swelling in the joints, and cartilage damage, in which these pathological conditions can persist for months or even years, in some cases [15]. Additionally, atypical cases involving complications associated with vision, cardiac, gastrointestinal, and neurological systems have been reported [17,18,19]. Moreover, Dengue (DENV) and/or Zika (ZIKV) patients co-infected by CHIKV have been described [20,21,22]. DENV and ZIKV are flaviviruses also transmitted by *Aedes* genus mosquitoes, which are associated with poverty and insufficient sanitation, being that DENV is a Neglected Tropical Disease (NTD), and CHIKV as well [23,24,25]. CHIKV is somewhat even more neglected than DENV [26,27].

Notwithstanding the high rates of CHIKV propagation, morbidity and mortality, there are no licensed vaccines or approved drugs to selectively treat this infectious disease [3,28,29]; although, research groups have reported the development of a new potential vaccine as a candidate for immunization against CHIKV [30]. Nowadays, the pharmacological treatment of CHIKV is limited to the antipyretic, analgesic, corticoid, and nonsteroidal anti-inflammatory drugs (NSAIDs) to relieve the typical symptoms [3,31].

The CHIKV genome comprises two open reading frames (ORFs), where a 5’ end ORF is capable of encoding four viral non-structural proteins (nsP1-nsP4), while a 3’ end ORF encodes viral structural proteins, which consist of capsid (C), two large enveloped glycoproteins (E1 and E2), and two accessory peptides (E3 and 6k) [32,33].

The nsP1 is involved in messenger RNA (mRNA) decoding and, via enzymatic activity of guanine-7-methyltransferase and guanylyltransferase, synthesis of viral negative single-stranded RNA [18,34,35,36]. In addition, the nsP2 has other essential enzymatic functions, which include RNA-helicase, nucleoside triphosphatase (NTPase), and RNA-dependent 5’-triphosphatase, where these are located in the *N*-terminal domain. In contrast, the *C*-terminal domain of nsP3 acts in part of the replicase and also as an accessory protein involved in the RNA synthesis [37,38,39]. Finally, the nsP4 acts as a RNA-dependent RNA-polymerase (RdRp) [18,38,40]. Differently, E1 and E2 proteins are responsible for spike glycoproteins in the surface from the viral particle, which facilitate the viral attachment to the susceptible host cells. The Ala/Val226 mutation in the E1 protein is associated with the generation of CHIKV-resistant strains [41,42]. Additionally, the Gly/Arg407 mutation in the E2 protein is responsible for generating CHIKV arbidol-resistant strains [43,44,45]. Typically, the CHIKV surface consists of 80 trimeric spikes composed of heterodimers of E1 and E2 proteins, in a lipid bilayer [46]. The CHIKV 6k is small, hydrophobic, and essential for the structural particle organization, in which it acts in the processing of sequence signaling of the E1 protein [46]. Nonetheless, the role of 6k in the CHIKV replication remains not fully understood [47].

**Figure 1:**
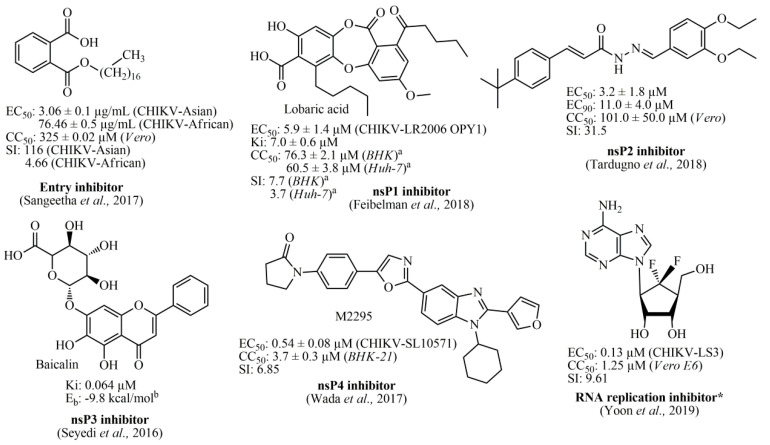
Best Chikungunya viral inhibitors reported in the literature and their corresponding targets. ^a^: values obtained after 24 hours; ^b^: Energy binding (E_b_) value obtained by using Auto Dock Vina software; *: Fluorinated adenosine analog was reported as an RNA replication inhibitor, in which its antiviral activity was associated with an indirect effect on viral methyltransferase (MTase) activity through the inhibition of host S-adenosyl-_L_-homocysteine (SAH) hydrolase.

Several in silico studies have identified that diverse chemical classes represent promising alternatives for the development of anti-CHIKV compounds, such as indoles [48], piperazines, benzimidazoles, pyrimidines [49], as well as phytocompounds [50]. Furthermore, studies involving organic synthesis have led to the obtainment of potential hit compounds, with low micromolar ranges. Among these, thiazolidine/rhodanine [51], peptidomimetic [52], thienopyrrole [53,54], triazolopyrimidinone [31], and adenosine analogs [55] have demonstrated excellent results. Therefore, one of the most promising chemical classes found in the literature is represented by acylhydrazone derivatives (and also acrylamide analogs), which have exhibited high activities with low cytotoxicity [56,57,58,59]. Essentially, the antiviral compounds can be categorized regarding their biological activity, being (*i*) CHIKV viral entry inhibitors [60,61]; (*ii*) nsP1 inhibitors [62]; (*iii*) nsP2 inhibitors [51,56,57,58,59]; (*iv*) nsP3 inhibitors [38]; (*v*) nsP4 inhibitors [63]; and (*vi*) viral RNA replication inhibitors [31,53,55,64,65]. In Figure 1, the best compounds found in the literature and their respective biological targets are illustrated. Finally, several other inhibitors targeting biological structures from CHIKV, as well as inhibitors with no specific targets can be found in the review manuscript by Silva-Júnior et al., (2017) [2].

**Figure 2:**
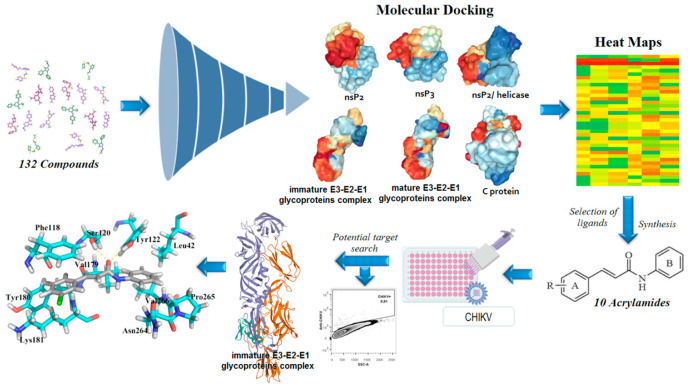
Workflow used to rationally develop acrylamides against the Chikungunya virus (CHIKV). In total, 132 compounds were analyzed toward nsP2 (Protein Data Bank (PDB): 3TRK), nsP2/helicase (PDB: 6JIM), nsP3 (PDB: 3GPO), immature E3-E2-E1 complex (PDB: 3N40), mature E3-E2-E1 complex (PDB: 3N41), and C (PDB: 5H23) proteins. Then, the most promising compounds were synthesized and screened. Lastly, the most active analog was docked into the E3-E2-E1 glycoproteins complex to identify the crucial amino acids involved in the ligand-target complex interactions.

In general, nsP2 inhibitors have been broadly explored by different research groups [51,56,57,58,59]. It is a cysteine protease that corresponds to approximately 20–30% of the viral particle, and also has four binding sites in its surface [1,52]. However, the binding site number 4 is considered the most important, since it contains the catalytic dyad, composed of Cys^1013^ and His^1083^ amino acids [49]. This cysteine protease has the potential to act as a nucleophile protein since the Cys^1013^ could be deprotonated by His^1083^, via an acid-base mechanism, at physiological pH [49,66]. Therefore, Michael’s acceptors represent an interesting alternative for developing anti-CHIKV compounds [56,58]. Additionally, the Trp^1084^ residue seems to perform an auxiliary role during the proteolytic activity of this protease [66]. As the global impacts of CHIKV and that the development of new antivirals is an unmet need, we aimed to develop new antiviral agents. The molecular docking of small flexible ligands toward macromolecules remains as the most broadly used in silico technique [67,68,69,70,71]. It is a stochastic method that uses scoring functions to find a minimum energy value, based on binding modes of ligands at the active site of macromolecules [72,73]. Essentially, it considers that hit compounds display high-affinity values forward their targets since they have favorable chemical groups for efficient interactions [69]. In this context, molecular docking was applied in this computer-guided study. Initially, virtual screening was performed in a dataset containing 132 compounds (including acrylamides and acylhydrazones) toward nsP2, nsP2/helicase, nsP3, immature and mature E3-E2-E1 glycoproteins complex, and C proteins. Meaningful FitScore values for acrylamide derivatives toward mature E3-E2-E1 glycoproteins complex were obtained by using molecular docking, which were analyzed by heat maps. Finally, an acrylamide analog exhibited a remarkable antiviral activity inhibiting the CHIKV infection in vitro, corroborating with our in silico results, suggesting that this compound acts by interaction with mature E3-E2-E1 glycoproteins complex and blocks the CHIKV attachment. Figure 2 presents the workflow including all the steps followed to perform this rational study.

## 2. Results and Discussion

### 2.1. Computer-Aided Drug Design

**Figure 3:**
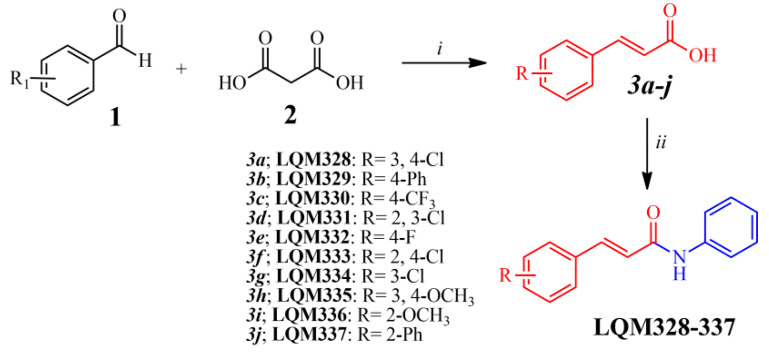
Synthetic route applied for the obtainment of acrylamide derivatives. Reagents and reactional conditions: (i) Pyridine, N-methylpiperazine (10 mol%), 24 hours, reflux; (ii) Aniline, dimethylformamide (DMF), 2-(1H-Benzotriazole-1-yl)-1,1’,3,3’-tetramethyluronium tetrafluoroborate (TBTU), diisopropylethylamine (DIPEA), 24 hours, at room temperature.

After the analysis of a dataset composed of 132 derivatives (including acrylamides and acylhydrazones) by molecular docking towards nsP2, nsP2/helicase, nsP3, immature and mature E3-E2-E1 glycoproteins complex, E, and C proteins from the CHIKV, it was identified that acrylamide derivatives could be more actives than acylhydrazones. In general, the 10 most favorable binding modes for each compound were generated by molecular docking. Moreover, it was verified that all molecules analyzed in this step demonstrated high affinity (FitScore) values forward the E3-E2-E1 glycoproteins complex (PDB ID: 3N41). Then, this observation was confirmed by using heat maps, in which a “hot-zone” was verified, concentrated on this molecular target. Thereafter, it was revealed that all acrylamides present FitScore values about 5–10 points higher than acylhydrazones. Additionally to this fact, some works have suggested that acylhydrazones and hydrazones could be associated with pan-assay interference scaffolds (PAINS), providing unreliable results in biological tests [74,75]. Combining this information and our virtual screening results, the top 10 most promising (FitScore ≥ 50.0) acrylamide derivatives (Figure 3) were selected for synthesis and biological evaluation in this study.

### 2.2. Chemistry

All the chemical intermediates (*3a-j*) were obtained by the reaction between corresponding aldehydes (1) and malonic acid (2), via Doebner–Knoevenagel condensation (Figure 3), with yields ranging from 45 to 94% [76,77]. Additionally, ^1^H Nuclear Magnetic Resonance (NMR) spectra revealed that the intermediates (*3a-j*) were obtained in an (*E*)-configuration, confirmed by large vinylic coupling constant (*J*) values, ranging from 15.8 to 16.1 Hz [77,78]. Moreover, ^1^H NMR analysis showed that the chemical shifts (*δ*) for the hydroxyl (OH) from the carboxylic acid can appear between 12.38 and 12.71 ppm. Subsequently, the acrylamide derivatives (LQM328-337) were obtained by the TBTU-coupling reaction (Figure 3), using diisopropylethylamine (DIPEA) as a catalyst base [79], with yields from 52 to 92%. For all these final compounds, the purity degree was determined by the HPLC technique, which resulted in purities ranging from 95.3 to 99.9%, with retention time (R_T_) between 3.07 and 3.88 min. The analysis of Fourier-Transform Infrared (FT-IR) spectra revealed three characteristic stretches (*v*) from these chemical molecules, ranging from 3240 to 3356 cm^−1^ for *v*(N‒H) bond; from 1658 to 1651 cm^−1^ for *v*(C=O) bond; also from 1620 to 1612 cm^−1^ and from 979 to 964 cm^−1^ *v*(C=C)_ene_ bond. Additionally, melting points (Mp) are uncorrected and they range from 113 to 227 °C. In the ^1^H NMR spectra of the acrylamides, it was observed that the amide (N‒H) signal ranges from 10.13 to 10.35 ppm. Equally to their intermediates, acrylamides were obtained in (*E*)-configuration, also confirmed by the vinylic coupling constant values, *J*. From ^13^C NMR spectra, the carbonyl (C=O) from the amide group has chemical shifts (*δ*) varying from 163.17 to 164.42 ppm. Additionally, elemental analyses (CHN) were only performed for the new acrylamides synthesized (LQM328, LQM331, and LQM337), in which their chemical compositions ranging from 61.95 to 83.52% for carbon, from 3.96 to 5.68% for hydrogen, and from 4.67 to 4.94% for nitrogen. For all acrylamides previously synthesized, the corresponding references were provided in order to compare the results obtained in this study (see Materials and Methods section). Finally, all these physicochemical and spectroscopic techniques were sufficient to unequivocally characterize the compounds synthesized in this work. All chromatograms, FT-IR, ^1^H and ^13^C NMR spectra are available in the Supplementary Material related to this manuscript.

### 2.3. Cell Viability and Antiviral Assays

**Figure 4:**
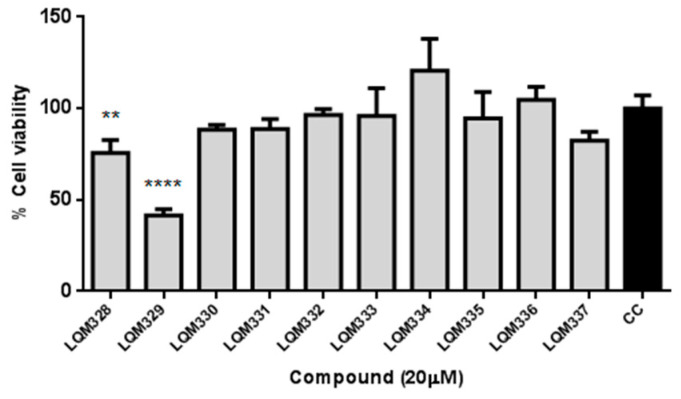
In vitro evaluation of cytotoxicity for synthesized acrylamides towards *Vero E6* cells. The cytotoxicity was performed in vitro for ten synthesized acrylamides (LQM328 to LQM337) towards *Vero E6* cells at 20 µM concentration by MTT assay after 48h. The expressed values are results from mean ± SD of triplicates at 20 µM concentration, analyzed after 48 hours. CC = cellular control. ** *p* ≤ 0.01; **** *p* ≤ 0.0001 versus CC.

The cytotoxicity was performed in vitro for the ten synthesized acrylamides (LQM328 to LQM337) towards *Vero E6* cells at 20 µM concentration by MTT assay [80]. As shown in Figure 4, only the LQM329 was highly cytotoxic, reducing the cell viability to less than 50% (41.5% ± 3.3) after 48h of culture, thus being removed for further analysis. Therefore, the screening of antiviral activity against CHIKV was performed for all other nine acrylamides.

**Figure 5:**
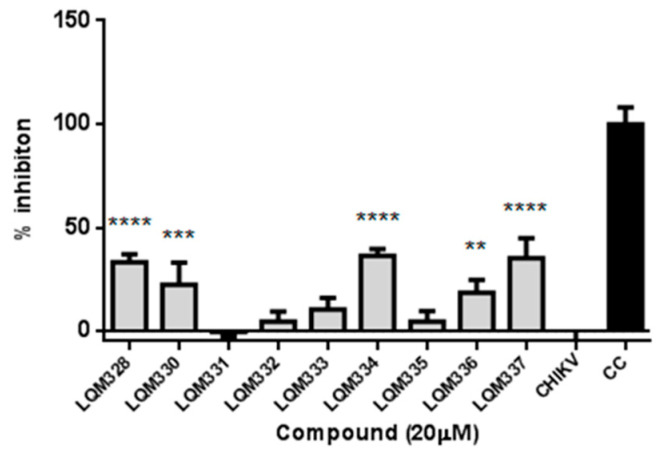
In vitro screening of anti-Chikungunya virus activity of acrylamides. The virus adsorption was performed for 2h followed by the addition of tested compounds at 20 µM concentration. The cell viability was assessed after 48h and the viral inhibition (%) was determined for each compound. The expressed values result from the mean ± SD of triplicates. CHIKV = CHIKV-infected untreated cells. CC = uninfected cellular control. ** *p* ≤ 0.01; *** *p* ≤ 0.001; **** *p* ≤ 0.0001 versus CHIKV.

Initially, the in vitro anti-CHIKV activity for the acrylamides was evaluated at a 20 µM concentration. For this purpose, CHIKV adsorption was performed in the *Vero E6* cells followed by the treatment with pre-selected compounds, and the cell viability was then assessed after 48h. As a result, significant viral inhibition was detected for the LQM328, LQM330, LQM334, LQM336, and LQM337 compounds (Figure 5).

**Figure 6:**
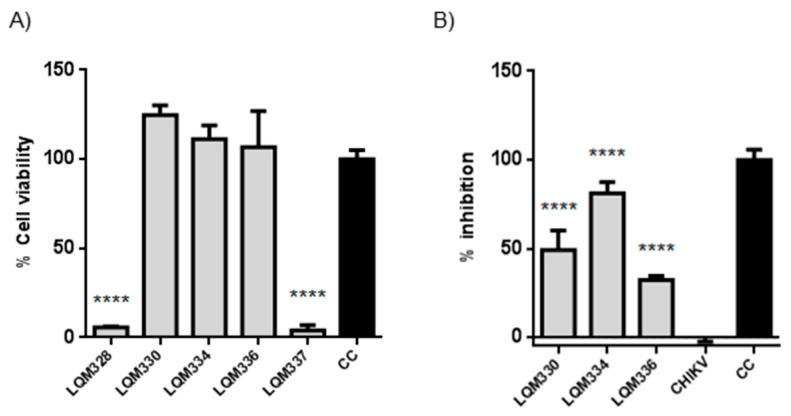
In vitro cytotoxicity and anti-Chikungunya virus results of acrylamides at 40 µM. (**A**) Evaluation of cytotoxicity for acrylamides in vitro towards *Vero E6* cells at 40 µM. The cytotoxicity was performed in vitro by MTT assay after 72h. **** *p* ≤ 0.0001 versus CC. (**B**) Assessment of anti-CHIKV activity of acrylamides at 40 µM. The virus adsorption was performed for 2 h followed by the addition of tested compounds. The cell viability was assessed after 72 h and the viral inhibition (%) was determined for each compound. **** *p* ≤ 0.0001 versus CHIKV. The expressed values result from the mean ± SD of triplicates. CHIKV = CHIKV-infected untreated cells. CC = uninfected cellular control.

To investigate the improvement in the antiviral activity of the compounds due to a higher concentration of the compounds, both in vitro cytotoxicity and anti-CHIKV assays were evaluated at 40 µM concentration, after 72h for the five most promising acrylamides (LQM328, LQM330, LQM334, LQM336, and LQM337). Although high cytotoxicity was detected for the LQM328 and LQM337, no toxicity was detected for LQM330, LQM334, and LQM336 at this concentration (Figure 6A). Regarding anti-CHIKV activity, a significant viral inhibition was detected for LQM330, LQM334, and LQM336, with the highest antiviral activity detected for the LQM334 (viral inhibition = 81.1% ± 6.4 for LQM334 vs. 49.1% ± 11.1 for LQM330, and 32.2% ± 2.4 for LQM336), as shown in Figure 6B.

### 2.4. Structure–Activity Relationship (SAR) Analysis

Posteriorly to the cell viability and antiviral assays, a structure–activity relationship (SAR) analysis was performed for this small series of acrylamide analogs (LQM328-337). As a rule, this will be discussed in the following sequence: electron-withdrawing; electron-donating; and aromatic ring substituents, considering the results at 20 and 40 µM, respectively.

Considering Figure 4 and Figure 5, the analog containing trifluoromethyl substituent at position 4 (LQM330) presents good cell viability (88.3% ± 2.8) and viral inhibition activity value of 22.4% ± 10.6. When this group is replaced with a fluorine atom results in an analog (LQM332) with better cell viability (96.4% ± 3.3), however, it abolishes the antiviral effect. Considering the chloro-containing derivatives, it is verified that the chlorinated 2,3-disubstituted analog (LQM331) demonstrates good cell viability; in contrast, it is completely inactive. When the chlorine atom at position 3 is shifted to position 4, an analog (LQM333) non-cytotoxic and inactive against CHIKV is obtained. Moreover, when the chlorine atom is shifted from position 2 to 3, a derivative (LQM328) which is slightly more cytotoxic is generated, presenting a cell viability value of 75.5% ± 7.2. Additionally, a small improvement in the antiviral activity is observed, with an inhibition value of 33.1% ± 3.9. However, it is important to note that LQM328 was highly cytotoxic at 40 µM. Finally, when the chlorine atom at position 4 is removed, with only one remaining chlorine atom at position 3 (LQM334), the most active acrylamide is obtained, with a viral inhibition value of 36.3% ± 3.3 at 20 µM and 81.1% ± 6.4, at 40 µM concentration. Regarding the acrylamides containing electron-donating substituents, the 3,4-disubstituted methoxyl compound (LQM335) showed no cytotoxicity and it was not active against CHIKV. Furthermore, its analog substituted only at position 2 (LQM336) shows a slight antiviral activity at 20 µM (18.5% ± 6.1). Finally, the acrylamide derivative containing a phenyl ring as a substituent at position 4 (LQM329) has high cytotoxicity, exhibiting a poor cell viability value of 41.5% ± 3.3. When this phenyl group is shifted to position 2 (LQM337), its cytotoxicity at 20 µM is abolished.

Concerning the results at 40 µM concentration (Figure 6), it is possible to verify that the 3,4-disubstituted chlorine compound (LQM328) becomes highly toxic (5.7% ± 0.6), in comparison with its results at 20 µM. In contrast, when the chlorine atom is removed from position 4, the cell viability is strongly increased (LQM334). Additionally, the analog containing a 4-trifluoromethyl substituent (LQM330) exhibited good cell viability. Similarly, the compound with a strong electron-donating group, such as 2-methoxyl substituent (LQM336), shows good cell viability. In contrast, a phenyl ring at position 2 (LQM337) showed high cytotoxicity. Interestingly, compounds presenting electron-withdrawing groups (LQM330 and 334) demonstrated better results in antiviral assays, with inhibition values of 49.1% ± 11.1 and 81.1% ± 6.4, respectively. Finally, LQM336 can be considered as a weak inhibitor against CHIKV, exhibiting an inhibition value of 32.2% ± 2.4, at 40 µM concentration.

**Figure 7:**
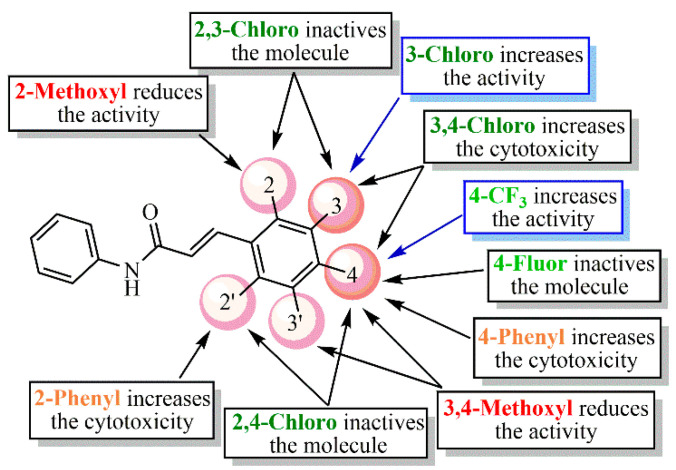
Summarization of structure–activity relationship (SAR) analysis for the acrylamides.

In brief, Figure 7 summarizes the SAR analysis for the acrylamide derivatives at 20 µM, since the number of compounds was higher than those at 40 µM concentration.

### 2.5. Intracellular Flow Cytometry Staining for CHIKV after Treatment with LQM334

**Figure 8:**
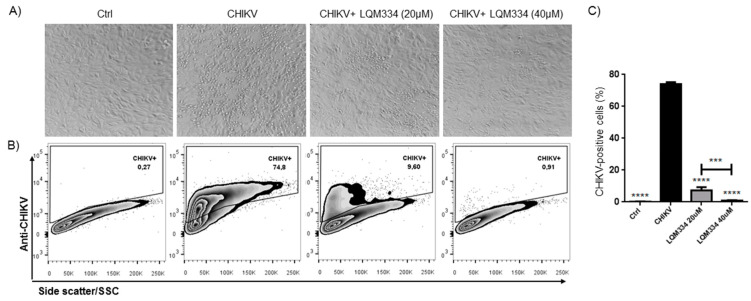
LQM334 inhibited the Chikungunya virus infection in vitro. In (**A**), Representative micrographs showing the cytopathogenic effect induced by the virus such as cell refringence changes and cell aggregates (200x magnification); In (**B**), Representative flow cytometry dot-plots of *Vero E6* cells infected (CHIKV) or uninfected (Control) with CHIKV. The cells were treated with LQM334 at 20 and 40 μM, respectively. The percentages of CHIKV-positive cells are shown; In (**C**), The expressed values are results from mean ± SD of triplicates. *** *p* ≤ 0.001; **** *p* ≤ 0.0001 versus untreated infected cells (CHIKV). CHIKV = CHIKV-infected untreated cells. Control/ctrl = uninfected cellular control.

In order to confirm the promising anti-CHIKV activity of LQM334, investigating its ability to inhibit the viral infection in *Vero E6* cells, the intracellular labeling of CHIKV was performed 48 h post-treatment and the percentage of CHIKV-positive cells was detected by flow cytometry. As shown in Figure 8A, LQM334 reduced the cytopathogenic effect induced by the virus compared to untreated cells (CHIKV). Moreover, LQM334 was able to significantly reduce the percentage of CHIKV-positive cells from 74.07% ± 1.19 to 7.38% ± 1.96 at 20 µM and to 0.88% ± 0.29 at 40 µM (Figure 8B,C). 

### 2.6. Molecular Docking Studies for LQM334

**Figure 9:**
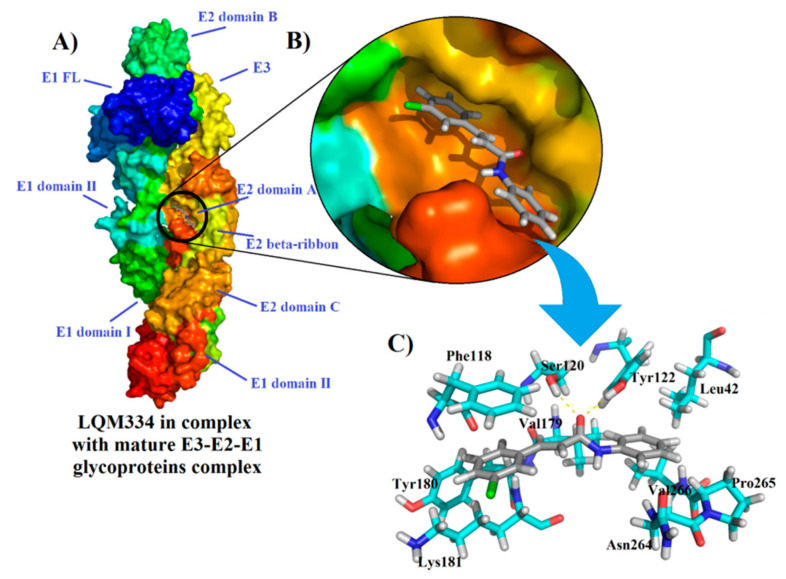
Interactions of LQM334 with mature E3-E2-E1 glycoproteins complex (PDB ID: 3N41) from the Chikungunya virus and its amino acids involved ligand-target complex. In (**A**), Overview of LQM334-mature E3-E2-E1 glycoproteins complex formation; In (**B**), Magnification showing the LQM334 into the central cleft from the glycoproteins complex; In (**C**), stick representations showing the amino acids involved in the complex formation with LQM334, in which yellow dots represent hydrogen bonding interactions.

After the obtainment of interesting results from the intracellular flow cytometry for the compound LQM334, a deep molecular docking analysis was performed throughout the possible six CHIKV targets, which were nsP2, nsP2/helicase, nsP3, immature and mature E3-E2-E1 glycoproteins complex, and C proteins. As a result, it was identified that LQM334 possibly binds more efficiently to the E2 domain A from the mature E3-E2-E1 glycoproteins complex (PDB: 3N41) (Figure 9A), exhibiting a FitScore value of 62.3293. Additionally, it is placed into the central cleft from the protein (Figure 9B). In order to obtain a FitScore parameter for comparison, the co-crystallized ligand from the mature E3-E2-E1 glycoproteins complex (a NAG molecule) was redocked using the same docking parameters, as described in methods’ section. To validate this approach, the root-mean-square deviation (RMSD) was used to evaluate how different the obtained docking orientation is from the corresponding co-crystallized pose of the NAG molecule. Concerning docking solutions, RMSD values allow to classify them as: (*a*) good solution when RMSD ≤ 2.0 Å; (*b*) acceptable solutions when RMSD is between 2.0 and 3.0 Å, and (*c*) bad solutions when RMSD ≥ 3.0 Å [81,82,83]. As a result, an RMSD value of 1.132 Å was obtained for the NAG redocking solution, suggesting a reliable docking protocol. Then, it was revealed that NAG had a FitScore value of 46.4102, suggesting that the LQM334 has a high affinity to this CHIKV target. According to Voss et al. (2010), the NAG molecule hydrophobically interacts with Lys^115^, Thr^116^, Phe^118^, Lys^181^, Leu^261^, Ala^262^, and Asn^263^ amino acid residues [84]. Regarding these interactions, LQM334 also interacts with Phe^118^ and Lys^181^ residues. Still, it hydrophobically interacts with Leu^42^, Val^179^, Tyr^180^, Asn^264^, Pro^265^, and Val^266^, while also interacting with Ser^120^ and Tyr^122^ amino acids, via hydrogen bonding interactions at distances of 2.07 and 2.1 Å, respectively (Figure 9C). Additionally, in silico studies involving E3-E2-E1 glycoproteins have been developed focusing on virtual screening of phenothiazines, bafilomycin [85], and FAD-approved antimicrobial agents, such as cefmenoxime, ceforanide, cefotetan, cefonicid sodium, and cefpiramide [86]. Recently, Song et al. (2019) [87] reported the crystal structures of the free mouse MXRA8 (mMXR8) receptor and the complex between human MXRA8 (hMXRA8) and the CHIKV E3-E2-E1 glycoproteins complex. From this study, the authors verified that the interaction between hMXRA8 and E3-E2-E1 glycoproteins complex occurs via 25 hydrophobic contacts, including Asn^264^ and Pro^265^ residues, as observed for LQM334 interactions. This fact reinforces that LQM334 possibly binds to an important binding site in the E3-E2-E1 glycoproteins complex and thus, it could suggest this molecule as a potential virus entry inhibitor.

## 3. Materials and Methods

### 3.1. Computational Details and Computer-Aided Drug Design

All in silico experiments involving molecular docking were performed in a Dell^®^ notebook, (Texas, USA), model 5500U, with an Intel^®^ Core^TM^ 4th generation *i*-7 processer, CPU 2.4 GHz, 16 GB RAM, and running at Windows^®^ 8.1 platform (Redmond, USA).

In total, 66 acrylamides and 66 acylhydrazones found in the literature were drawn, converted into three-dimensional structures, and energetically minimized by the application of the semi-empiric method Austin Model 1 (AM1), by using ArgusLab^®^ software (Richland, USA), v. 4.0.1 (http://www.arguslab.com) [88]. Three-dimensional structures of nsP2 (PDB: 3TRK) [89], nsP2/helicase (PDB: 6JIM) [90], nsP3 (PDB: 3GPO) [91], immature E3-E2-E1 complex (PDB: 3N40) [84], mature E3-E2-E1 complex (PDB: 3N41) [84], and C (PDB: 5H23) [92] proteins were obtained at the Research Collaboratory for Structural Bioinformatics Protein Data Bank (RCSB PDB, San Diego, USA), website (https://www.rcsb.org). Pretreatment of these macromolecules and molecular docking simulations were performed using GOLD^®^ software (Cambridge, UK), v. 5.8.1 (https://www.ccdc.cam.ac.uk/solutions/csd-discovery/components/gold/) [93]. Co-crystallized molecules from targets’ structures were used for redocking in order to obtain FitScore cutoff values to build the virtual protocol. Subsequently, all these co-crystallized molecules were removed, including water molecules and ions. Then, H-bond acceptor and donor atoms were assumed as solvent accessible. Additionally, all four different scoring functions from GOLD^®^ software were employed, being Chemical Piecewise Linear Potential (CHEMPLP), GoldScore, ChemScore, and Astex Statistical Potential (ASP). Finally, the correlation coefficient (*r^2^*) for each scoring function was determined by the comparison between the redocked co-crystallized molecules and ligands in this study, acrylamides and acylhydrazones, using Microsoft Excel^®^ 2010 to obtain useful heat maps (red and green mean hot and cold, respectively). Furthermore, all energetic contributions and interactions (H-bond, hydrophobic, and van der Waals) were individually analyzed. All illustrations were generated by using PyMol^®^ software, v. 0.99 (https://pymol.org/2/). Lastly, all procedures performed in this study are in accordance with recently published works by our research team [94,95,96,97,98,99,100].

### 3.2. Reagents and Solvents

All starting reagents and solvents were purchased from Merck/Sigma-Aldrich^®^ Company (St. Louis, MO, USA), and they were commercial products of high purity (>98%). Additionally, the solvents used in reactions and column chromatography were subjected to rotary evaporation before use to remove impurities. Finally, in high-performance liquid chromatography (HPLC) experiments, methanol HPLC degree from Tedia^®^ High Purity Solvents Company (Fairfield, OH, USA) was used as eluent.

### 3.3. Chemical Characterization and Apparatus

For the intermediate products (3a-j), information about yields and physical aspects was provided. Additionally, these were only characterized by hydrogen Nuclear Magnetic Resonance (^1^H NMR) since they are not new in the literature. For the final products (LQM328-LQM337), information about yields and physical aspects, purity, retention time (R_T_), melting point (Mp) or degradation point (Dp), Fourier-Transform Infrared (FT-IR) spectra, ^1^H and ^13^C NMR spectra, and elemental analyses (CHN) were provided, since most of the final products are completely new in the literature. Finally, for the acrylamides previously synthesized, the corresponding references were also provided in this section.

### 3.4. High-Performance Liquid Chromatography—(HPLC)

For purity degree (%) and R_T_ determination for the final compounds, a Shimadzu^®^ (Kyoto, Japan) chromatograph was used, model SIL-20AHT, utilizing a Luna^®^ 5 µm C18(2) 100 Å column, with dimensions of 250 × 4.6 mm, and wavelength (*λ*) of 254 nm. All HPLC runs were performed using methanol HPLC degree (≥99%) as a mobile phase. Moreover, some parameters were established, such as (a) sample concentration of 1 mg/mL; (b) flow of 1 mL/min; (c) run time of 15 min; and (d) injection volume of 5 µL. Finally, the retention times (R_T_) were computed in minutes (min) and absorbance, in mili-absorbance unities (mAU) [101].

### 3.5. Melting Point Determination

All melting point (Mp) for final compounds were determined by using an MSTecnopon^®^ (Piracicaba, Brazil), model PFMII Digital, with maximum temperature at 330 °C, utilizing glass capillaries containing the samples. Initially, 40 °C was admitted as a starting temperature and then a temperature increase by 1 °C/min was allowed. In some cases, Mp due to the degradation of the sample was not observed. Thus, the degradation point (Dp) was computed. Finally, all Mp or Dp are uncorrected and were assumed in a range of 1 °C between the values [102].

### 3.6. Fourier-Transform Infrared Spectroscopy–(FT-IR)

All FT-IR spectra were obtained using a spectrophotometer from Shimadzu^®^ (Kyoto, Japan), model IRPrestige-21, employing the attenuated total reflectance (ATR) method, in the range from 4000 to 400 cm^−1^ [103,104]. All spectra were treated by using Shimadzu IRsolution^®^ software, version 1.50, 2008. Finally, all bond stretches (v) and angle deformations (δ) for the main functional group from the final compounds (acrylamide moiety) were computed in transmittance (T%) and wavenumber (cm^−1^) [105].

### 3.7. ^1^H and ^13^C Nuclear Magnetic Resonance Spectroscopy–(NMR)

All spectra of ^1^H and ^13^C NMR were obtained utilizing a Bruker^®^ equipment (Billerica, USA), model UltraShield 600 MHz. Besides, deuterated methanol (MeOD), chloroform (CDCl_3_), and dimethylsulfoxide (DMSO-*d*_6_) were used as analytical solvents, depending on the samples’ solubility. In total, it was admitted scans’ number of 16 and 1024 for hydrogen and carbon nuclei, respectively. All chemical shifts were computed in part *per* millions (ppm). Additionally, coupling constants (J) were determined in hertz (Hz). Moreover, signal multiplicities were attributed as singlet (s), broad singlet (br s), doublet (d), double-doublets (dd), triplet (t), triplet of doublets (td), quartet (q), and multiplet (m) [78,106]. Finally, all NMR spectra were treated and analyzed by using the academic licensed Bruker TopSpin^®^ software, version 4.0.7, 2019.

### 3.8. Elemental Analysis (CHN)

Samples containing 1–2 mg of LQM328, LQM331, and LQM337 were placed into tin capsules for solids, specific for elemental analyses. All determinations were carried out in a Perkin Elmer^®^ equipment, model CHNS/O Analyzer 2400 series II. For combustion and reduction columns, the temperatures of 950 and 640 °C were used, respectively. Gas pressures for O_2_ e He were admitted as 140 and 105 KPa, respectively. Additionally, a combustion column filling-time of 30 s was assumed. Finally, a total run-time of 5 min for each sample was allowed. These procedures are adaptations from works previously reported [107,108,109].

### 3.9. Synthesis of Cinnamic Acid and Acrylamide Derivatives

#### 3.9.1. General Procedures for the Obtainment of Cinnamic Acids (3a-j)

In general, an adaptation of methods described by Luo and collaborators (2015) was used [110], through a Knoevenagel condensation Doebner modification reaction [77]. In a bottom flask (50 mL) containing 6 mL pyridine, the corresponding aldehydes (1.0 eq.) were added. Subsequently, malonic acid (1.1 eq.) was also added into the solution. After 15 min under reflux and stirring, *N*-methyl piperazine (10 mol%) was added as a catalyst base. Then, the reactional mixture was kept at these conditions overnight. After the reaction completion (verified by TLC), 10 mL distilled water was added to the crude mixture, providing a white powder precipitated. The heterogeneous mixture was refrigerated (2 °C) by 30 min. Posteriorly, the mixture was stirred for 10 min and, then, 15 mL concentrated HCl (37%) was added into the flask until the pH of 1. Finally, the resulting precipitated was filtered and washed by distilled water (2 × 50 mL), affording the corresponding cinnamic acids.

*(E)-3-(3,4-Dichlorophenyl)acrylic acid **(3a)***

Yield: 60%. Aspect: white amorphous powder. ^1^H NMR (600 MHz, MeOD) *δ* (ppm): 6.53 (*d*, 1H, CH_ene_, *J* = 15.9 Hz), 7.53–7.55 (*m*, 2H, CH_Ar_), 7.6 (*d*, 1H, CH_ene_, *J* = 15.9 Hz), 7.79 (*s*, 1H, CH_Ar_).

*(E)-3-([1,1’-Biphenyl]-4-yl)acrylic acid **(3b)***

Yield: 94%. Aspect: yellow amorphous powder. ^1^H NMR (600 MHz, DMSO-*d*_6_) *δ* (ppm): 6.58 (*d*, 1H, CH_ene_, *J* = 15.9 Hz), 7.38 (*t*, 1H, CH_Ar_, *J* = 7.3 Hz), 7.47 (*t*, 2H, CH_Ar_, *J* = 7.6 Hz), 7.64 (*d*, 1H, CH_ene_, *J* = 15.9 Hz), 7.7–7.72 (*m*, 4H, CH_Ar_), 7.78 (*d*, 1H, CH_Ar_, *J* = 8.2 Hz), 12.44 (*br s*, 1H, OH).

*(E)-3-(4-(Trifluoromethyl)phenyl)acrylic acid **(3c)***

Yield: 45%. Aspect: white amorphous powder. ^1^H NMR (600 MHz, MeOD) *δ* (ppm): 6.48 (*d*, 1H, CH_ene_, *J* = 16.0 Hz), 7.56 (*d*, 1H, CH_Ar_, *J* = 8.0 Hz), 7.57 (*d*, 1H, CH_ene_, *J* = 16.0 Hz), 7.65 (*d*, 1H, CH_Ar_, *J* = 8.0 Hz), 8.05 (*d*, 1H, CH_Ar_, *J* = 8.0 Hz).

*(E)-3-(2,3-Dichlorophenyl)acrylic acid **(3d)***

Yield: 78%. Aspect: white amorphous powder. ^1^H NMR (600 MHz, DMSO-*d*_6_) *δ* (ppm): 5.71 (*d*, 1H, CH_ene_, *J* = 15.9 Hz), 6.53 (*t*, 1H, CH_Ar_, *J* = 8.0 Hz), 6.77 (*dd*, 1H, CH_Ar_, *J* = 8.0 and 1.5 Hz), 6.92 (*dd*, 1H, CH_Ar_, *J* = 7.8 and 1.2 Hz), 7.24 (*d*, 1H, CH_ene_, *J* = 15.9 Hz).

*(E)-3-(4-Fluorophenyl)acrylic acid **(3e)***

Yield: 80%. Aspect: white amorphous powder. ^1^H NMR (600 MHz, DMSO-*d*_6_) *δ* (ppm): 6.5 (*d*, 1H, CH_ene_, *J* = 16.0 Hz), 7.25 (*t*, 2H, CH_Ar_, *J* = 8.8 Hz), 7.59 (*d*, 1H, CH_ene_, *J* = 15.9 Hz), 7.76–7.78 (*m*, 2H, CH_Ar_), 12.41 (*br s*, 1H, OH).

*(E)-3-(2,4-Dichlorophenyl)acrylic acid **(3f)***

Yield: 65%. Aspect: white amorphous powder. ^1^H NMR (600 MHz, DMSO-*d*_6_) *δ* (ppm): 6.62 (*d*, 1H, CH_ene_, *J* = 15.9 Hz), 7.45 (*dd*, 1H, CH_Ar_, *J* = 8.4 and 2.1 Hz), 7.7 (*d*, 1H, CH_Ar_, *J* = 2.0 Hz), 7.79 (*d*, 1H, CH_ene_, *J* = 15.9 Hz), 7.94 (*d*, 1H, CH_Ar_, *J* = 8.5 Hz), 12.71 (*br s*, 1H, OH).

*(E)-3-(3-Chlorophenyl)acrylic acid **(3g)***

Yield: 90%. Aspect: gray amorphous powder. ^1^H NMR (600 MHz, DMSO-*d*_6_) *δ* (ppm): 6.62 (*d*, 1H, CH_ene_, *J* = 16.0 Hz), 7.42–7.47 (*m*, 2H, CH_Ar_), 7.57 (*d*, 1H, CH_ene_, *J* = 15.9 Hz), 7.67 (*d*, 1H, CH_Ar_, *J* = 7.3 Hz), 7.78 (*s*, 1H, CH_Ar_), 12.53 (*br s*, 1H, OH).

*(E)-3-(3,4-Dimethoxyphenyl)acrylic acid **(3h)***

Yield: 75%. Aspect: white amorphous powder. ^1^H NMR (600 MHz, CDCl_3_) *δ* (ppm): 3.93 (*s*, 6H, CH_3_), 6.33 (*d*, 1H, CH_ene_, *J* = 15.8 Hz), 6.89 (*d*, 1H, CH_Ar_, *J* = 8.3 Hz), 7.08 (*d*, 1H, CH_Ar_, *J* = 1.8 Hz), 7.15 (*dd*, 1H, CH_Ar_, *J* = 8.3 and 1.8 Hz), 7.74 (*d*, 1H, CH_ene_, *J* = 15.8 Hz).

*(E)-3-(2-Methoxyphenyl)acrylic acid **(3i)***

Yield: 90%. Aspect: white amorphous powder. ^1^H NMR (600 MHz, MeOD) *δ* (ppm): 3.89 (*s*, 3H, CH_3_), 6.5 (*d*, 1H, CH_ene_, *J* = 16.1 Hz), 6.96 (*t*, 1H, CH_Ar_, *J* = 7.3 Hz), 7.03 (*d*, 1H, CH_ene_, *J* = 8.4 Hz), 7.37 (*td*, 1H, CH_Ar_, *J* = 8.4, 1.5 and 1.3 Hz), 7.56 (*dd*, 1H, CH_Ar_, *J* = 7.32 and 1.6 Hz), 7.97 (*d*, 1H, CH_ene_, *J* = 16.1 Hz).

*(E)-3-([1,1’-Biphenyl]-2-yl)acrylic acid **(3j)***

Yield: 85%. Aspect: gray amorphous powder. ^1^H NMR (600 MHz, DMSO-*d*_6_) *δ* (ppm): 6.49 (*d*, 1H, CH_ene_, *J* = 15.9 Hz), 7.31 (*d*, 2H, CH_Ar_, *J* = 6.9 Hz), 7.36 (*d*, 1H, CH_Ar_, *J* = 7.4 Hz), 7.42–7.45 (*m*, 2H, CH_Ar_), 7.48–7.51 (*m*, 4H, CH_Ar_), 7.9 (*d*, 1H, CH_Ar_, *J* = 7.4 Hz), 12.38 (*br s*, 1H, OH).

#### 3.9.2. General Procedures for the Obtainment of Acrylamides (LQM328–LQM337)

In general, an adaptation of methods described by Tanja and collaborators (2019) was used [79]. Initially, aniline (1.0 eq.) was added into a bottom flask (50 mL) containing 5 mL dimethylformamide (DMF) as the solvent. The corresponding cinnamic acid derivatives (1.1 eq.) were posteriorly added. Then, 2-(1*H*-Benzotriazole-1-yl)-1,1’,3,3’-tetramethyluronium tetrafluoroborate—TBTU (1.0 eq.) was also added to the solution under stirring by 10 min. Subsequently, *N, N*’-diisopropylethylamine—DIPEA (3.5 eq.) was used added as a catalyst base. The reactional mixture remained under stirring and room temperature by 48 h. Then, 20 mL saturated NaHCO_3_ solution (15 mL) was added to the crude mixture, providing a precipitate. This heterogeneous mixture was stirred for 15 min and, subsequently, the residual solid was filtered and washed with a saturated NaHCO_3_ solution (3 × 10 mL) and, then, distilled water (3 × 25 mL), yielding the corresponding acrylamide derivatives. In some cases, it was necessary to further purifications. Lastly, these analogs were recrystallized from an acetone/water mixture (1:2), filtered and washed by distilled water (3 × 10 mL), providing pure products.

*(E)-3-(3,4-Dichlorophenyl)-N-phenylacrylamide **(LQM328)***

Yield: 55%. Aspect: yellow amorphous powder. Purity: 99.4%. R_T_: 3.88 min. Mp. 145–146 °C. FT-IR (cm^−1^): 3240 *v*(N‒H), 1651 *v*(C=O), 1612 and 979 *v*(C=C)_ene_. ^1^H NMR (600 MHz, DMSO-*d*_6_) *δ* (ppm): 6.89 (*d*, 1H, CH_ene_, *J* = 15.7 Hz), 7.08 (*t*, 1H, CH_Ar_, *J* = 7.3 Hz), 7.34 (*t*, 2H, CH_Ar_, *J* = 7.9 Hz), 7.57 (*d*, 1H, CH_ene_, *J* = 15.7 Hz), 7.63 (*dd*, 1H, CH_Ar,_ *J* = 8.3 and 1.9 Hz), 7.7 (*t*, 3H, CH_Ar_, *J* = 8.3 Hz), 7.9 (*d*, 1H, CH_Ar_, *J* = 1.9 Hz), 10.24 (*br s*, 1H, NH). ^13^C NMR (150 MHz, DMSO-*d*_6_) *δ* (ppm): 119.74, 123.99, 125.15, 127.86, 129.29, 130.06, 131.61, 132.22, 132.35, 136.17, 137.9, 139.55, 163.5. CHN_calculed_ (%) for C_15_H_11_Cl_2_NO: C 61.67, H 3.8, N 4.79. CHN_found_ (%): C 61.95, H 3.96, N 4.94.

*(E)-3-([1,1’-Biphenyl]-4-yl)-N-phenylacrylamide **(LQM329)***

Yield: 52%. Aspect: white amorphous powder. Purity: 97.7%. R_T_: 3.52 min. Mp: 226–227 °C. FT-IR (cm^−1^): 3309 *v*(N‒H), 1658 *v*(C=O), 1620 and 972 *v*(C=C)_ene_. ^1^H NMR (600 MHz, DMSO-*d*_6_) *δ* (ppm): 6.89 (*d*, 1H, CH_ene_, *J* = 15.6 Hz), 7.07 (*t*, 1H, CH_Ar_, *J* = 7.3 Hz), 7.34 (*t*, 2H, CH_Ar_, *J* = 7.8 Hz), 7.39 (*t*, 1H, CH_Ar_, *J* = 7.3 Hz), 7.48 (*t*, 2H, CH_Ar_, *J* = 7.6 Hz), 7.63 (*d*, 1H, CH_ene_, *J* = 15.6 Hz), 7.71 (*d*, 6H, CH_Ar_, *J* = 7.8 Hz), 7.76 (*d*, 2H, CH_Ar_, *J* = 8.2 Hz), 10.23 (*br s*, 1H, NH). ^13^C NMR (150 MHz, DMSO-*d*_6_) *δ* (ppm): 119.72, 122.75, 123.83, 127.1, 127.67, 128.34, 128.83, 129.27, 129.49, 134.34, 139.76, 140.1, 141.75, 164.02.

Reference: Carissimi (1959) [111].

*(E)-N-Phenyl-3-(4-(trifluoromethyl)phenyl)acrylamide **(LQM330)***

Yield: 87%. Aspect: white amorphous powder. Purity: 96.5%. R_T_: 3.23 min. Mp: 154–155 °C. FT-IR (cm^−1^): 3356 *v*(N‒H), 1651 *v*(C=O), 1620 and 972 *v*(C=C)_ene_. ^1^H NMR (600 MHz, DMSO-*d*_6_) *δ* (ppm): 6.97 (*d*, 1H, CH_ene_, *J* = 15.7 Hz), 7.09 (*t*, 1H, CH_Ar_, *J* = 7.3 Hz), 7.35 (*t*, 2H, CH_Ar_, *J* = 7.8 Hz), 7.67 (*d*, 1H, CH_ene_, *J* = 15.7 Hz), 7.7 (*d*, 2H, CH_Ar_, *J* = 7.8 Hz), 7.81 (*d*, 2H, CH_Ar_, *J* = 8.4 Hz), 7.85 (*d*, 2H, CH_Ar_, *J* = 8.2 Hz), 10.31 (*br s*, 1H, NH). ^13^C NMR (150 MHz, DMSO-*d*_6_) *δ* (ppm): 119.76, 124.03, 125.47, 125.63, 126.36, 128.79, 129.31, 138.84, 139.29, 139.56, 163.49.

Reference: Guo et al. (2012) [112].

*(E)-3-(2,3-Dichlorophenyl)-N-phenylacrylamide **(LQM331)***

Yield: 90%. Aspect: white amorphous powder. Purity: 99.3%. R_T_: 3.48 min. Mp: 212–213 °C. FT-IR (cm^−1^): 3255 *v*(N‒H), 1651 *v*(C=O), 1620 and 964 *v*(C=C)_ene_. ^1^H NMR (600 MHz, DMSO-*d*_6_) *δ* (ppm): 6.89 (*d*, 1H, CH_ene_, *J* = 15.5 Hz), 7.08 (*t*, 1H, CH_Ar_, *J* = 7.08 Hz), 7.34 (*t*, 2H, CH_Ar_, *J* = 7.8 Hz), 7.46 (*t*, 1H, CH_Ar_, *J* = 7.9 Hz), 7.69 (*d*, 2H, CH_Ar_, *J* = 7.9 Hz), 7.72 (*d*, 2H, CH_Ar_, *J* = 7.8 Hz), 7.87 (*d*, 1H, CH_ene_, *J* = 15.6 Hz), 10.35 (*br s*, 1H, NH). ^13^C NMR (150 MHz, DMSO-*d*_6_) *δ* (ppm): 119.82, 124.13, 126.82, 127.31, 129.11, 129.32, 131.69, 131.73, 133.08, 135.69, 135.76, 139.45, 163.17. CHN_calculed_ (%) for C_15_H_11_Cl_2_NO: C 61.67, H 3.8, N 4.79. CHN_found_ (%): C 62.21, H 3.75, N 4.67.

*(E)-3-(4-Fluorophenyl)-N-phenylacrylamide **(LQM332)***

Yield: 81%. Aspect: white amorphous powder. Purity: 99.4%. R_T_: 3.16 min. Mp: 135–136 °C. FT-IR (cm^−1^): 3309 *v*(N‒H), 1658 *v*(C=O), 1620 and 964 *v*(C=C)_ene_. ^1^H NMR (600 MHz, DMSO-*d*_6_) *δ* (ppm): 6.77 (*d*, 1H, CH_ene_, *J* = 15.7 Hz), 7.06 (*t*, 1H, CH_Ar_, *J* = 7.3 Hz), 7.28 (*t*, 2H, CH_Ar_, *J* = 8.7 Hz), 7.33 (*t*, 2H, CH_Ar_, *J* = 7.8 Hz), 7.58 (*d*, 1H, CH_ene_, *J* = 15.7 Hz), 7.67–7.7 (*m*, 4H, CH_Ar_), 10.2 (*br s*, 1H, NH). ^13^C NMR (150 MHz, DMSO-*d*_6_) *δ* (ppm): 116.39, 119.72, 122.69, 123.83, 129.25, 130.33, 131.85, 139.39, 139.71, 162.51, 163.92.

Reference: Sethiya et al. (2019) [113].

*(E)-3-(2,4-Dichlorophenyl)-N-phenylacrylamide **(LQM333)***

Yield: 92%. Aspect: white amorphous powder. Purity: 99.4%. R_T_: 3.63 min. Mp: 180–181 °C. FT-IR (cm^−1^): 3278 *v*(N‒H), 1651 *v*(C=O), 1620 and 964 *v*(C=C)_ene_. ^1^H NMR (600 MHz, DMSO-*d*_6_) δ (ppm): 6.9 (*d*, 1H, CH_ene_, *J* = 15.6 Hz), 7.08 (*t*, 1H, CH_Ar_, *J* = 7.3 Hz), 7.34 (*t*, 2H, CH_Ar_, *J* = 7.8 Hz), 7.53 (*dd*, 1H, CH_Ar_, *J* = 8.4 and 1.8 Hz), 7.7 (*d*, 2H, CH_Ar_, *J* = 8.0 Hz), 7.74 (*d*, 1H, CH_Ar_, *J* = 2.0 Hz), 7.77 (*d*, 1H, CH_Ar_, *J* = 8.5 Hz), 7.79 (*d,* 1H, CH_ene_, *J* = 15.6 Hz), 10.32 (*br s*, 1H, NH). ^13^C NMR (150 MHz, DMSO-*d*_6_) *δ* (ppm): 119.78, 124.09, 126.56, 128.61, 129.31, 129.41, 129.99, 132.12, 134.6, 134.68, 135.2, 139.47, 163.25.

Reference: Qiu and Zhang (2014) [114].

*(E)-3-(3-Chlorophenyl)-N-phenylacrylamide **(LQM334)***

Yield: 81%. Aspect: brown amorphous powder. Purity: 99.9%. R_T_: 3.58 min. Mp: 123–124 °C. FT-IR (cm^−1^): 3278 *v*(N‒H), 1651 *v*(C=O), 1620 and 964 *v*(C=C)_ene_. ^1^H NMR (600 MHz, DMSO-*d*_6_) *δ* (ppm): 6.89 (*d*, 1H, CH_ene_, *J* = 15.7 Hz), 7.08 (*t*, 1H, CH_Ar_, *J* = 7.3 Hz), 7.34 (*t*, 2H, CH_Ar_, *J* = 7.8 Hz), 7.47–7.49 (*m*, 2H, CH_Ar_), 7.58 (*d*, 1H, CH_ene_, *J* = 15.7 Hz), 7.59–7.61 (*m*, 1H, CH_Ar_), 7.69–7.1 (*m*, 3H, CH_Ar_), 10.23 (*br s*, 1H, NH). ^13^C NMR (150 MHz, DMSO-*d*_6_) *δ* (ppm): 119.73, 123.95, 124.56, 126.61, 127.84, 129.28, 129.81, 131.3, 134.2, 137.54, 138.94, 139.6, 163.64.

Reference: Frei et al. (2017) [115].

*(E)-3-(3,4-Dimethoxyphenyl)-N-phenylacrylamide **(LQM335)***

Yield: 72%. Aspect: white amorphous powder. Purity: 99.5%. R_T_: 3.07 min. Mp: 130–131 °C. FT-IR (cm^−1^): 3309 *v*(N‒H), 1651 *v*(C=O), 1620 and 964 *v*(C=C)_ene_. ^1^H NMR (600 MHz, DMSO-*d*_6_) *δ* (ppm): 3.8 (*s*, 3H, CH_3_), 3.82 (*s*, 3H, CH_3_), 6.71 (*d*, 1H, CH_ene_, *J* = 15.6 Hz), 7.01 (*d*, 1H, CH_Ar_, *J* = 8.2 Hz), 7.05 (*t*, 1H, CH_Ar_, *J* = 7.3 Hz), 7.19 (*dd*, 1H, CH_Ar_, *J* = 8.2 and 1.5 Hz), 7.22 (*d*, 1H, CH_Ar_, *J* = 1.5 Hz), 7.33 (*t*, 2H, CH_Ar_, *J* = 7.8 Hz), 7.53 (*d*, 1H, CH_ene_, *J* = 15.6 Hz), 7.7 (*d*, 2H, CH_Ar_, *J* = 7.8 Hz), 10.13 (*br s*, 1H, NH). ^13^C NMR (150 MHz, DMSO-*d*_6_) *δ* (ppm): 55.88, 56.04, 110.48, 112.25, 119.57, 120.35, 122.25, 123.63, 127.96, 129.24, 139.91, 140.73, 149.4, 150.86, 164.31.

Reference: Araújo-Vilges et al. (2017) [116].

*(E)-3-(2-Methoxyphenyl)-N-phenylacrylamide **(LQM336)***

Yield: 90%. Aspect: white amorphous powder. Purity: 99.2%. R_T_: 3.11 min. Mp: 136–137 °C. FT-IR (cm^−1^): 3286 *v*(N‒H), 1651 *v*(C=O), 1620 and 972 *v*(C=C)ene. ^1^H NMR (600 MHz, DMSO-*d*_6_) *δ* (ppm): 3.88 (*s*, 3H, CH_3_), 6.88 (*d*, 1H, CH_ene_, *J* = 15.8 Hz), 7.01 (*t*, 1H, CH_Ar_, *J* = 7.4 Hz), 7.05 (*t*, 1H, CH_Ar_, *J* = 7.3 Hz), 7.09 (*d*, 1H, CH_Ar_, *J* = 8.3 Hz), 7.32 (*t*, 2H, CH_Ar_, *J* = 7.7 Hz), 7.39 (*t*, 1H, CH_Ar_, *J* = 7.7 Hz), 7.57 (*d*, 1H, CH_Ar_, *J* = 7.4 Hz), 7.7 (*d*, 2H, CH_Ar_, *J* = 7.9 Hz), 7.81 (*d*, 1H, CH_ene_, *J* = 15.7 Hz), 10.18 (*br s*, 1H, NH). ^13^C NMR (150 MHz, DMSO-*d*_6_) *δ* (ppm): 56.09, 112.25, 119.5, 121.23, 123.1, 123.54, 123.72, 128.69, 129.24, 131.66, 135.78, 139.58, 158.2, 164.42.

Reference: Ittyerah and Pandya (1941) [117], and Yamamori et al. (2004) [118].

*(E)-3-([1,1’-Biphenyl]-2-yl)-N-phenylacrylamide **(LQM337)***

Yield: 84%. Aspect: brown amorphous powder. Purity: 95.3%. R_T_: 3.38 min. Mp: 194–195 °C. FT-IR (cm^−1^): 3255 *v*(N‒H), 1651 *v*(C=O), 1620 and 972 *v*(C=C)_ene_. ^1^H NMR (600 MHz, DMSO-*d*_6_) *δ* (ppm): 6.84 (*d*, 1H, CH_ene_, *J* = 15.5 Hz), 7.05 (*t*, 1H, CH_Ar_, *J* = 7.3 Hz), 7.3–7.34 (*m*, 4H, CH_Ar_), 7.43–7.46 (*m*, 2H, CH_Ar_), 7.49–7.51 (*m*, 5 H, CH_Ar_ and CH_ene_), 7.66 (*d*, 2H, CH_Ar_, *J* = 7.85 Hz), 7.78–7.8 (*m*, 1H, CH_Ar_), 10.22 (*br s*, 1H, NH). ^13^C NMR (150 MHz, DMSO-*d*_6_) *δ* (ppm): 119.65, 123.63, 123.83, 126.73, 128.01, 128.46, 128.88, 129.24, 130.06, 130.25, 130.91, 132.91, 139.02, 139.67, 140.18, 142.6, 163.85. CHN_calculed_ (%) for C_21_H_17_NO: C 84.25, H 5.72, N 4.68. CHN_found_ (%): C 83.52, H 5.68, N 4.81.

### 3.10. Cell Viability Assay

Briefly, *Vero E6* cells were plated at 2 × 10^4^ cells/well in 96-well microplate and cultured in at 37 °C and 5% CO_2_ atmosphere. Confluent cells monolayers were cultured in DMEM-low glucose (Sigma-Aldrich^®^, St. Louis, MO, USA) with 2% fetal bovine serum (Gibco) and antibiotic antimycotic solution (Gibco), for 48h or 72h in the presence of acrylamides at 20 or 40 µM. The cell viability was then evaluated using MTT (3-(4,5-Dimethyl-2-thiazolyl)-2,5-diphenyl-2H-tetrazolium (Sigma-Aldrich^®^, St. Louis, MO, USA) cytotoxic assay [80]. Therefore, the MTT solution was added at a final concentration of 0.5 mg/mL followed by incubation for 3h. The culture medium was removed and 150 µL of dimethyl sulfoxide (DMSO) was added to each well leading to formazan solubilization. The value of blank control absorbance (only the used culture medium in the absence of cells) was subtracted from all samples. The absorbance of each well was measured at a 492 nm wavelength and the percentage of cell viability was calculated as follows:

*Cell viability (%) = [sample absorbance/average of cell control absorbance] × 100.* (1)

### 3.11. In Vitro Antiviral Assay

Initially, a serial dilution of CHIKV stock was performed and the viral dilution that has been reduced cell viability at least 80% was used in antiviral assays (data not shown). After that, *Vero E6* cells were plated at 2 × 10^4^ cells/well in a 96-well microplate and maintained at 37 °C and 5% CO_2_ atmosphere until reaching the confluence of ~80–90%. The virus adsorption was then performed by incubating the cells with CHIKV diluted 1:200 in DMEM-low glucose medium/2% bovine fetal serum for 2 h with homogenization every 15 min. Thereafter, the medium was removed and the cellular monolayers were washed with phosphate-buffered saline, and several synthesized acrylamides were added at 20 or 40 µM. The cell viability was assessed after 48 or 72 h by MTT cell viability assay as previously described (topic 3.9). The percentage of viral inhibition was calculated as follows:

*Inhibition (%) = [sample absorbance − average of viral control absorbance/average of* (2)

*cellular control absorbance − average of viral control absorbance] × 100.*

### 3.12. Intracellular Flow Cytometry Staining for CHIKV

The antiviral activity of the LQM334 compound was confirmed by intracellular flow cytometry staining [119]. Briefly, after CHIKV adsorption in *Vero E6* cells for 2 h, the medium was removed, the cell monolayer was washed with PBS. The LQM334 compound was added at 20 or 40 μM concentrations and the cells were maintained at 37 °C/5% CO_2_ atmosphere for 48h. The cells were detached by using a trypsin/EDTA solution and submitted to fixation and permeabilization using the BD Cytofix/ Cytoperm^TM^ Fixation/Permeabilization solution kit (BD Biosciences^®^, San Jose, CA, USA) according to the manufacturer’s recommendations. Subsequently, the cells were incubated with an anti-CHIKV monoclonal antibody (1:50; A54Q clone; Invitrogen, Carlsbad, CA, USA) for 1h at 4 °C. The cells were washed with the BD Perm Wash solution (BD Biosciences^®^, San Jose, CA, USA) and then incubated with the goat anti-mouse IgG (H + L) cross-adsorbed secondary antibody conjugated with Alexa Fluor 488 (1:200; Invitrogen) for 1h at 4 °C. A total of 20,000 events were acquired in the BD FACS Canto^TM^ II flow cytometer (BD Biosciences^®^, San Jose, CA, USA) and the results were analyzed by using FlowJo^TM^ v. 10 software.

### 3.13. Statistical Analysis

The statistical analyses were performed in the GraphPad Prism^®^ v.6.0 software (San Diego, CA, USA) using One-Way ANOVA followed by Dunnett multiple comparison tests, and the *p* ≤ 0.05 was considered statistically significant.

## 4. Conclusions

A dataset of 132 compounds (including acrylamides and acylhydrazones) was built for a virtual protocol. Then, the computer-aided drug design protocol applied towards six CHIKV targets was able to identify 10 promising acrylamides to be synthesized and biologically evaluated. In the cytotoxicity assay, nine of 10 synthesized compounds presented a cell viability higher than 75%, at 20 µM concentration, with promising results in regards to the challenge in infected *Vero E6* cells with CHIKV. As a result, the compound LQM334 was found to be the most active analog, even though in a higher concentration, it kept the cell viability and viral inhibition. Additionally, the intracellular flow cytometry staining demonstrated that LQM334 inhibited cell infection by the CHIKV. Regarding the antiviral activity of LQM334, the deep molecular docking analysis pointed to a possible virus target for LQM334. In this regard, it was identified that LQM334 preferably interacts with the E2 domain A from the mature E3-E2-E1 glycoproteins complex from CHIKV. Additionally, it displays hydrogen bonding interactions with Ser^120^ and Tyr^122^ amino acid residues. Therefore, our virtual pipeline pointed out a potential inhibitor of E3-E2-E1 glycoproteins complex from CHIKV with antiviral activity. It suggests that the medicinal chemistry of CHIKV should be more explored in order to provide more information about the most relevant chemical classes of compounds for designing new antiviral agents. Finally, this work represents the emergence of a new potential E3-E2-E1 glycoprotein complex inhibitor, which is in continuous development and several structural optimizations are being performed currently in order to identify new potent candidates with low toxicity. Concerning all of the results for LQM334, it is possible to suggest that this acrylamide analog could be used as a promising anti-CHIKV scaffold for the design of new antiviral agents in the future.

## Supplementary Materials

The following are available online at https://www.mdpi.com/1424-8247/13/7/141/s1, **Figure S1**. *^1^H NMR spectrum of (E)-3-(3,4-Dichlorophenyl)acrylic acid* (3a), **Figure S2**. *^1^H NMR spectrum of (E)-3-([1,1’-Biphenyl]-4-yl)acrylic acid* (3b), **Figure S3**. *^1^H NMR spectrum of (E)-3-(4-(Trifluoromethyl)phenyl)acrylic acid* (3c), **Figure S4**. *^1^H NMR spectrum of (E)-3-(2,3-Dichlorophenyl)acrylic acid* (3d), **Figure S5**. *^1^H NMR spectrum of (E)-3-(4-Fluorophenyl)acrylic acid* (3e), **Figure S6**. *^1^H NMR spectrum of (E)-3-(2,4-Dichlorophenyl)acrylic acid* (3f), **Figure S7**. *^1^H NMR spectrum of (E)-3-(3-Chlorophenyl)acrylic acid* (3g), **Figure S8.** *^1^H NMR spectrum of (E)-3-(3,4-Dimethoxyphenyl)acrylic acid* (3h), **Figure S9**. *^1^H NMR spectrum of (E)-3-(2-Methoxyphenyl)acrylic acid* (3i), **Figure S10**. *^1^H NMR spectrum of (E)-3-([1,1’-Biphenyl]-2-yl)acrylic acid* (3j), **Figure S11**. *HPLC chromatogram of (E)-3-(3,4-Dichlorophenyl)-N-phenylacrylamide* (LQM328), **Figure S12***. FT-IR spectrum of (E)-3-(3,4-Dichlorophenyl)-N-phenylacrylamide* (LQM328), **Figure S13**. *¹H NMR spectrum of (E)-3-(3,4-Dichlorophenyl)-N-phenylacrylamide* (LQM328), **Figure S14**. *¹³C NMR spectrum of (E)-3-(3,4-Dichlorophenyl)-N-phenylacrylamide* (LQM328), **Figure S15.** *HPLC chromatogram of (E)-3-([1,1’-Biphenyl]-4-yl)-N-phenylacrylamide* (LQM329), **Figure S16**. *FT-IR spectrum of* *(E)-3-([1,1’-Biphenyl]-4-yl)-N-phenylacrylamide* (LQM329), **Figure S17**. *¹H NMR spectrum of (E)-3-([1,1’-Biphenyl]-4-yl)-N-phenylacrylamide (LQM329),* **Figure S18***. ¹³C NMR spectrum of (E)-3-([1,1’-Biphenyl]-4-yl)-N-phenylacrylamide (LQM329),* **Figure S19.** *HPLC chromatogram of (E)-N-Phenyl-3-(4-(trifluoromethyl)phenyl)acrylamide (LQM330),* **Figure S20.** *FT-IR spectrum of (E)-N-Phenyl-3-(4-(trifluoromethyl)phenyl)acrylamide (LQM330),* **Figure S21.** *¹H NMR spectrum of (E)-N-Phenyl-3-(4-(trifluoromethyl)phenyl)acrylamide (LQM330),* **Figure S22.** *¹³C NMR spectrum of (E)-N-Phenyl-3-(4-(trifluoromethyl)phenyl)acrylamide (LQM330),* **Figure S23***. HPLC chromatogram of (E)-3-(2,3-Dichlorophenyl)-N-phenylacrylamide (LQM331),* **Figure S24.** *FT-IR spectrum of (E)-3-(2,3-Dichlorophenyl)-N-phenylacrylamide (LQM331),* **Figure S25.** *¹H NMR spectrum of (E)-3-(2,3-Dichlorophenyl)-N-phenylacrylamide (LQM331),* **Figure S26***. ¹³C NMR spectrum of (E)-3-(2,3-Dichlorophenyl)-N-phenylacrylamide (LQM331),* **Figure S27***. HPLC chromatogram of (E)-3-(4-Fluorophenyl)-N-phenylacrylamide (LQM332),* **Figure S28.** *FT-IR spectrum of* *(E)-3-(4-Fluorophenyl)-N-phenylacrylamide (LQM332),* **Figure S29.** *¹H NMR spectrum of (E)-3-(4-Fluorophenyl)-N-phenylacrylamide (LQM332),* **Figure S30.** *¹³C NMR spectrum of (E)-3-(4-Fluorophenyl)-N-phenylacrylamide (LQM332),* **Figure S31.** *HPLC chromatogram of (E)-3-(2,4-Dichlorophenyl)-N-phenylacrylamide (LQM333),* **Figure S32.** *FT-IR spectrum of (E)-3-(2,4-Dichlorophenyl)-N-phenylacrylamide (LQM333),* **Figure S33***. ¹H NMR spectrum of (E)-3-(2,4-Dichlorophenyl)-N-phenylacrylamide (LQM333),* **Figure S34.** *¹³C NMR spectrum of (E)-3-(2,4-Dichlorophenyl)-N-phenylacrylamide (LQM333),* **Figure S35.** *HPLC chromatogram of (E)-3-(3-Chlorophenyl)-N-phenylacrylamide (LQM334),* **Figure S36***. FT-IR spectrum of* *(E)-3-(3-Chlorophenyl)-N-phenylacrylamide (LQM334),* **Figure S37.** *¹H NMR spectrum of (E)-3-(3-Chlorophenyl)-N-phenylacrylamide (LQM334),* **Figure S38.** *¹³C NMR spectrum of (E)-3-(3-Chlorophenyl)-N-phenylacrylamide (LQM334),* **Figure S39.** *HPLC chromatogram of (E)-3-(3,4-Dimethoxyphenyl)-N-phenylacrylamide (LQM335),* **Figure S40***. FT-IR spectrum of (E)-3-(3,4-Dimethoxyphenyl)-N-phenylacrylamide (LQM335),* **Figure S41.** *¹H NMR spectrum of (E)-3-(3,4-Dimethoxyphenyl)-N-phenylacrylamide (LQM335),* **Figure S42***. ¹³C NMR spectrum of (E)-3-(3,4-Dimethoxyphenyl)-N-phenylacrylamide (LQM335),* **Figure S43.** *HPLC chromatogram of (E)-3-(2-Methoxyphenyl)-N-phenylacrylamide (LQM336),* **Figure S44.** *FT-IR spectrum of (E)-3-(2-Methoxyphenyl)-N-phenylacrylamide (LQM336),* **Figure S45.** *¹H NMR spectrum of (E)-3-(2-Methoxyphenyl)-N-phenylacrylamide (LQM336),* **Figure S46***. ¹³C NMR spectrum of (E)-3-(2-Methoxyphenyl)-N-phenylacrylamide (LQM336),* **Figure S47.** *HPLC chromatogram of (E)-3-([1,1’-Biphenyl]-2-yl)-N-phenylacrylamide (LQM337),* **Figure S48.** *FT-IR spectrum of* *(E)-3-([1,1’-Biphenyl]-2-yl)-N-phenylacrylamide (LQM337),* **Figure S49.** *¹H NMR spectrum of (E)-3-([1,1’-Biphenyl]-2-yl)-N-phenylacrylamide (LQM337),* **Figure S50.** *¹³C NMR spectrum of (E)-3-([1,1’-Biphenyl]-2-yl)-N-phenylacrylamide (LQM337)*.
