# Supplementary material for: Computer-Aided Design, Synthesis, and Antiviral Evaluation of Novel Acrylamides as Potential Inhibitors of E3-E2-E1 Glycoproteins Complex from Chikungunya Virus
Source: Pharmaceuticals (Basel). 2020 Jun 30;13(7):141. doi: 10.3390/ph13070141 (PMC7407227; doi:10.3390/ph13070141)

Computer-Aided Design, Synthesis, and Antiviral Evaluation of Novel Acrylamides as Potential Inhibitors of E3-E2-E1 Glycoproteins Complex from Chikungunya Virus

Gabriel Felipe Silva Passos [1], Matheus Gabriel Moura Gomes [1], Thiago Mendonça de Aquino [2], João Xavier de Araújo-Júnior [1], Stephannie Janaína Maia Souza [3], João Pedro Monteiro Cavalcante [3], Elane Conceição dos Santos [3], Ênio José Bassi [3], and Edeildo Ferreira da Silva-Júnior [1, 2 *]

[1] Laboratory of Medicinal Chemistry, Pharmaceutical Sciences Institute, Federal University of Alagoas, Maceió, Brazil; e-mails: gabrielfelipepassos@gmail.com (G.F.S.P.); matheus_gabriel199@hotmail.com (M.G.M.G.); jotaaraujo2004@gmail.com (J.X.A.J.)

[2] Center of Analysis and Research in Nuclear Magnetic Resonance, Chemistry and Biotechnology Institute, Federal University of Alagoas, Maceió, Brazil; e-mail: thiago.aquino@iqb.ufal.br (T.M.A.)

[3] Immunoregulation Research Group, Laboratory of Research in Virology and Immunology, Institute of Biological and Health Sciences, Federal University of Alagoas, Maceió, Brazil; e-mails: stephanniemaia92@gmail.com (S.J.M.S.); j.p.monteirocavalcante@gmail.com (J.P.M.C.); elane.santos@icbs.ufal.br (E.C.S.); enio.bassi@icbs.ufal.br (Ê.J.B.)

* Correspondence: edeildo.junior@iqb.ufal.br; Tel.: +55-87-9-9610-8311 (E.F.S.J.)

* SUPPLEMENTARY MATERIAL *

Figure S1. *^{1}H NMR spectrum of (E)-3-(3,4-Dichlorophenyl)acrylic acid* **(3a)**

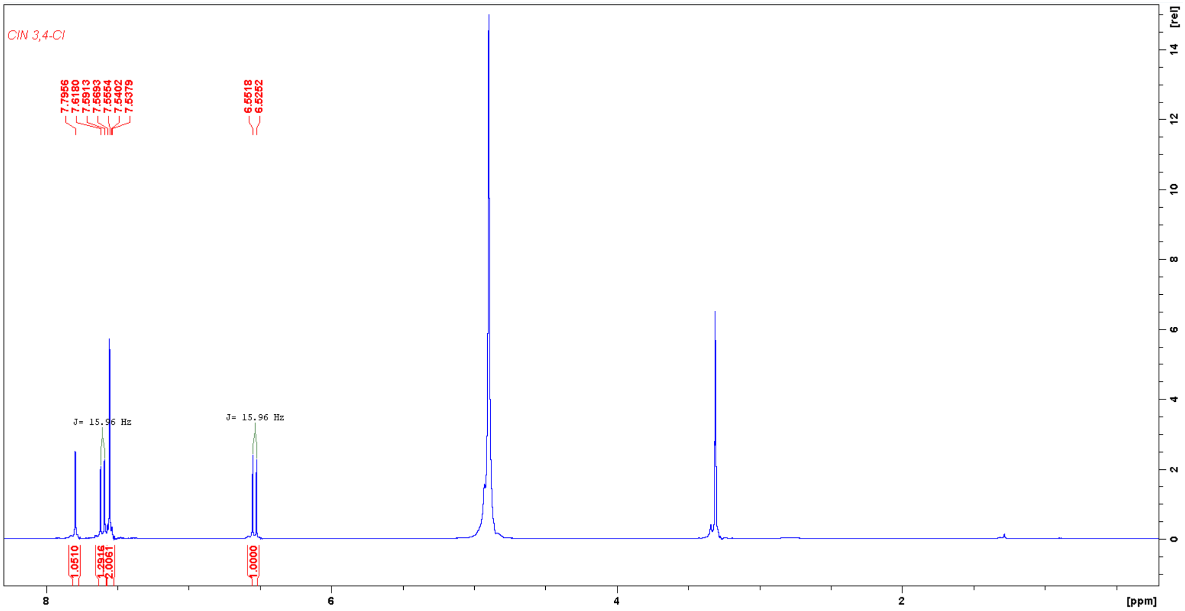

Figure S2. *^{1}H NMR spectrum of (E)-3-([1,1'-Biphenyl]-4-yl)acrylic acid* **(3b)**

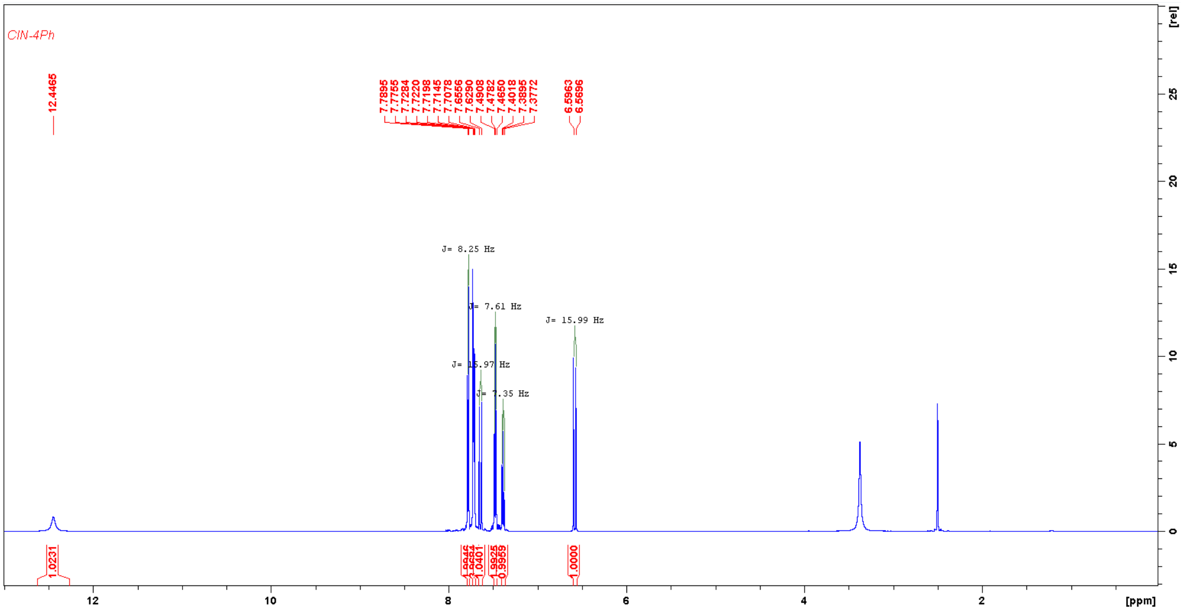

Figure S3. *^{1}H NMR spectrum of (E)-3-(4-(Trifluoromethyl)phenyl)acrylic acid* **(3c)**

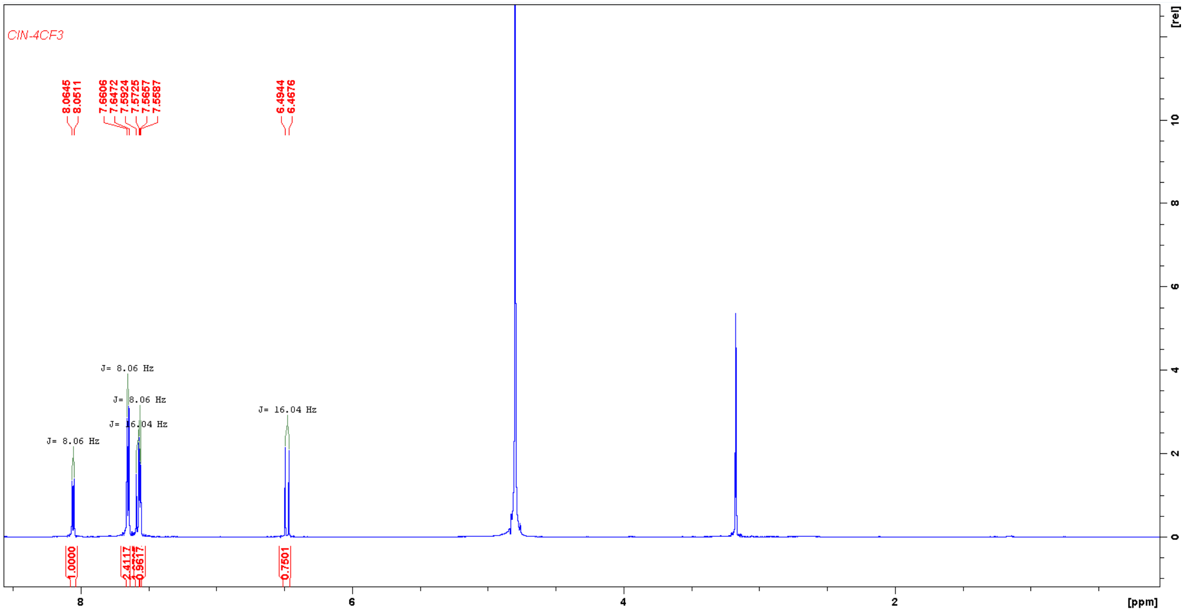

Figure S4. *^{1}H NMR spectrum of (E)-3-(2,3-Dichlorophenyl)acrylic acid* **(3d)**

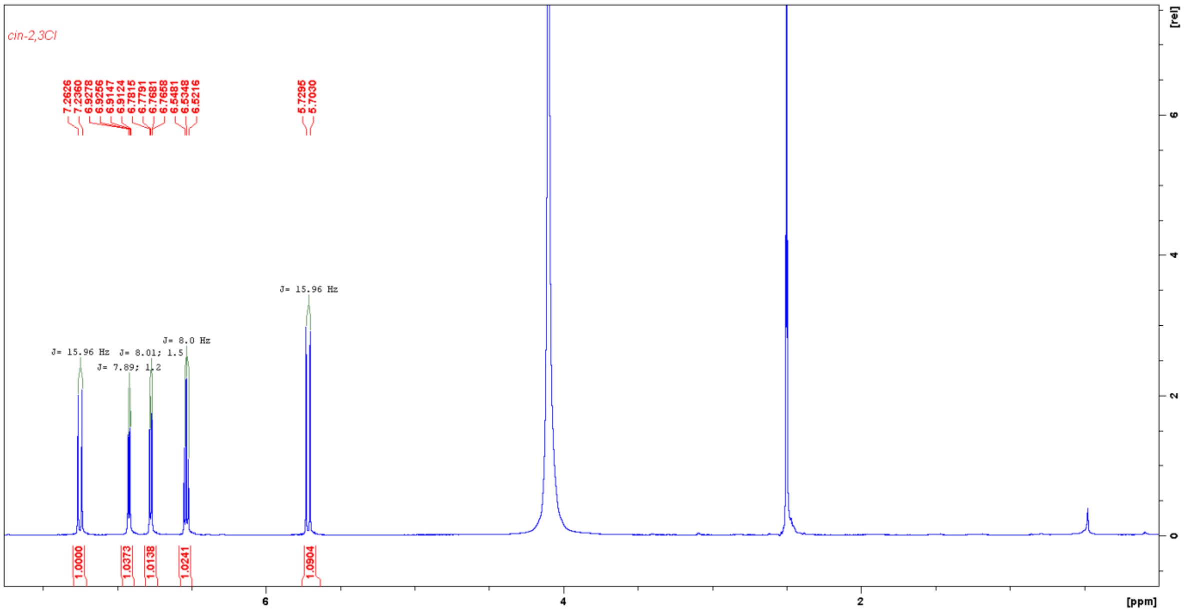

Figure S5. *^{1}H NMR spectrum of (E)-3-(4-Fluorophenyl)acrylic acid* **(3e)**

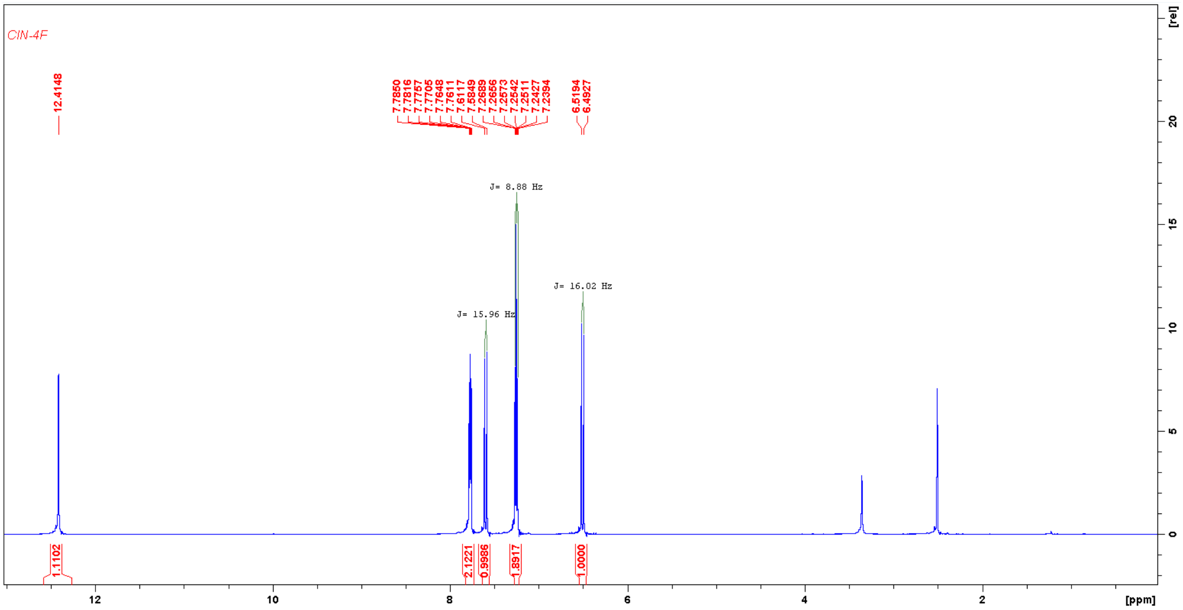

Figure S6. *^{1}H NMR spectrum of (E)-3-(2,4-Dichlorophenyl)acrylic acid* **(3f)**

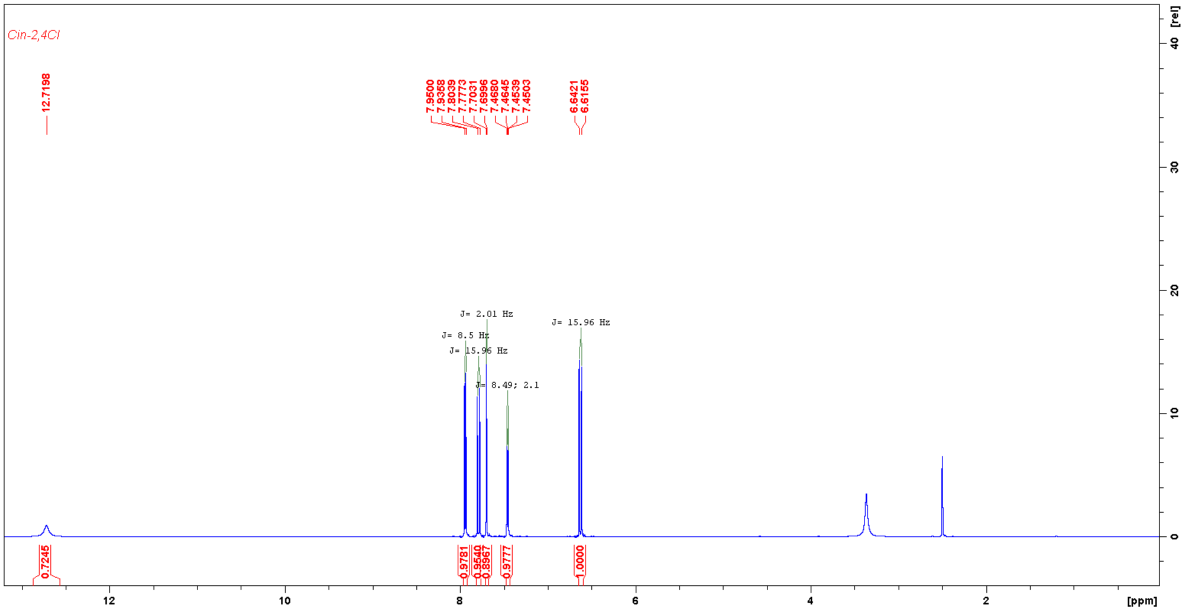

Figure S7. *^{1}H NMR spectrum of (E)-3-(3-Chlorophenyl)acrylic acid* **(3g)**

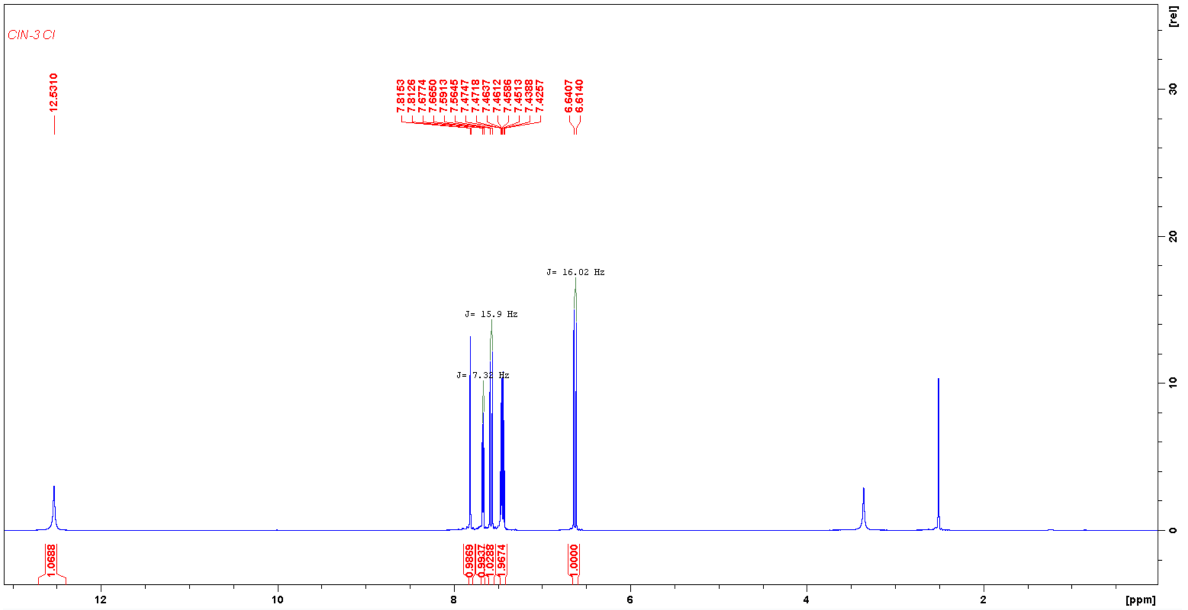

Figure S8. *^{1}H NMR spectrum of (E)-3-(3,4-Dimethoxyphenyl)acrylic acid* **(3h)**

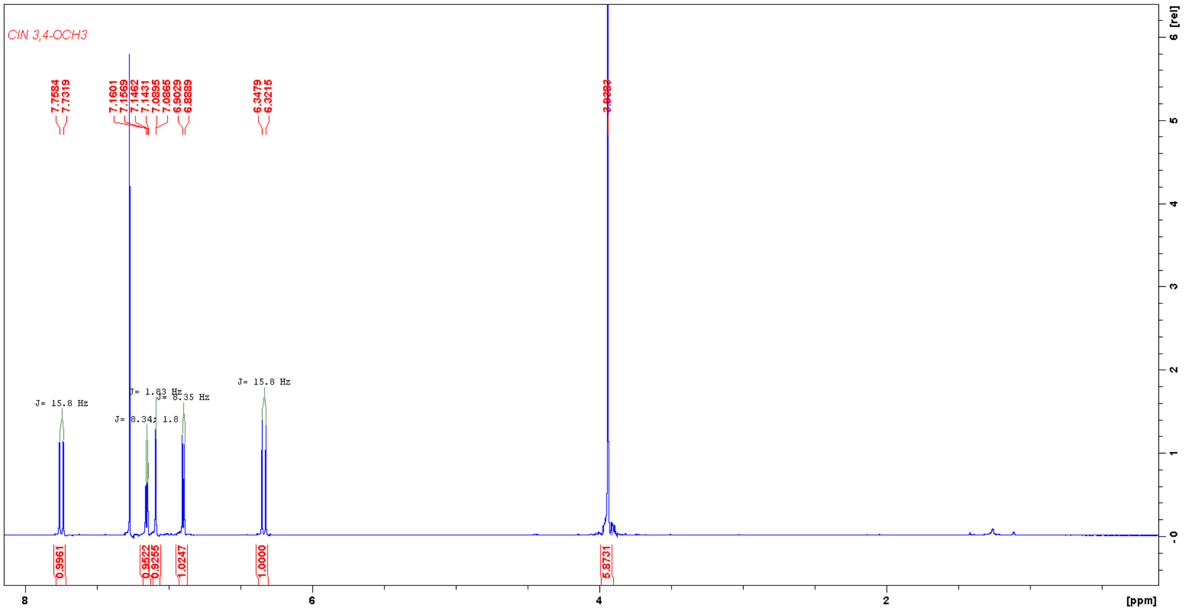

Figure S9. *^{1}H NMR spectrum of (E)-3-(2-Methoxyphenyl)acrylic acid* **(3i)**

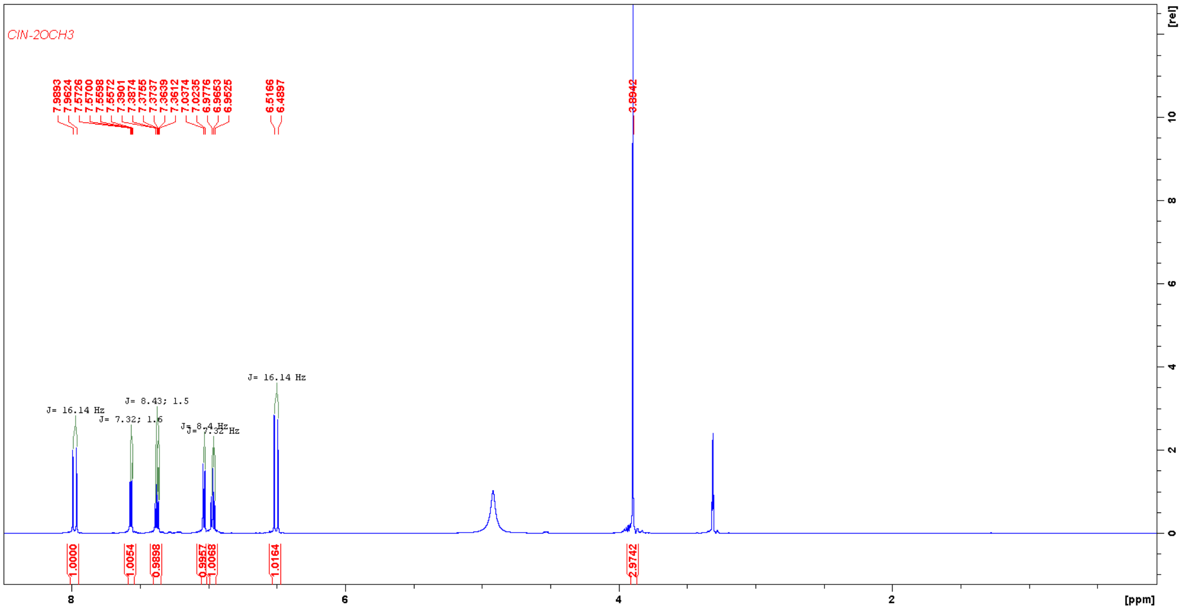

Figure S10. *^{1}H NMR spectrum of (E)-3-([1,1'-Biphenyl]-2-yl)acrylic acid* **(3j)**

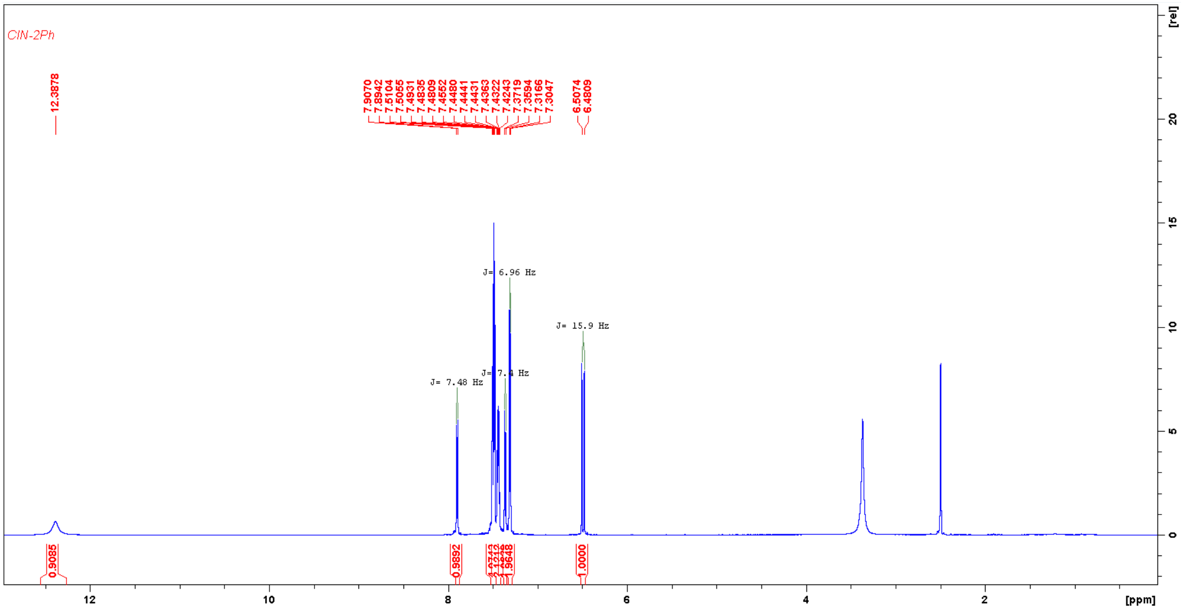

Figure S11. *HPLC chromatogram of (E)-3-(3,4-Dichlorophenyl)-N-phenylacrylamide* **(LQM328)**

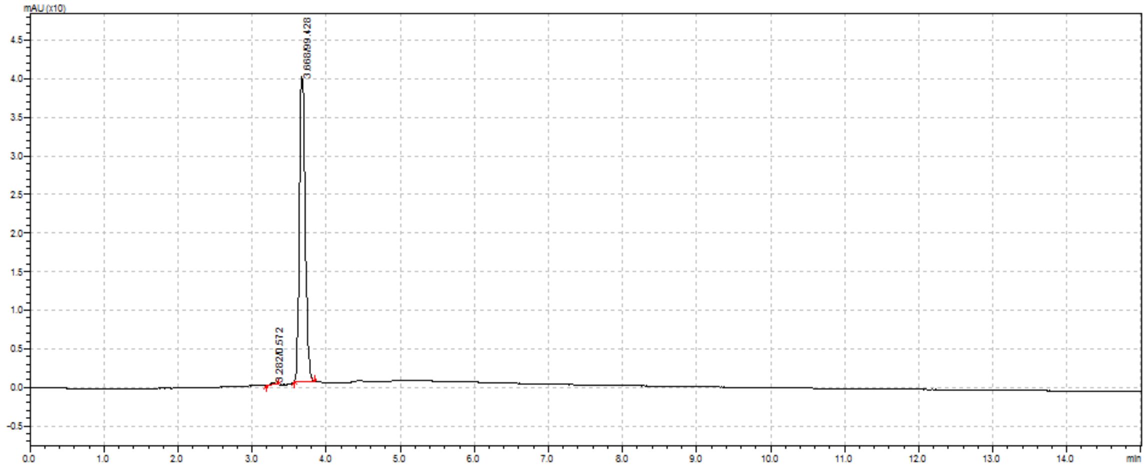

Figure S12. *FT-IR spectrum of (E)-3-(3,4-Dichlorophenyl)-N-phenylacrylamide* **(LQM328)**

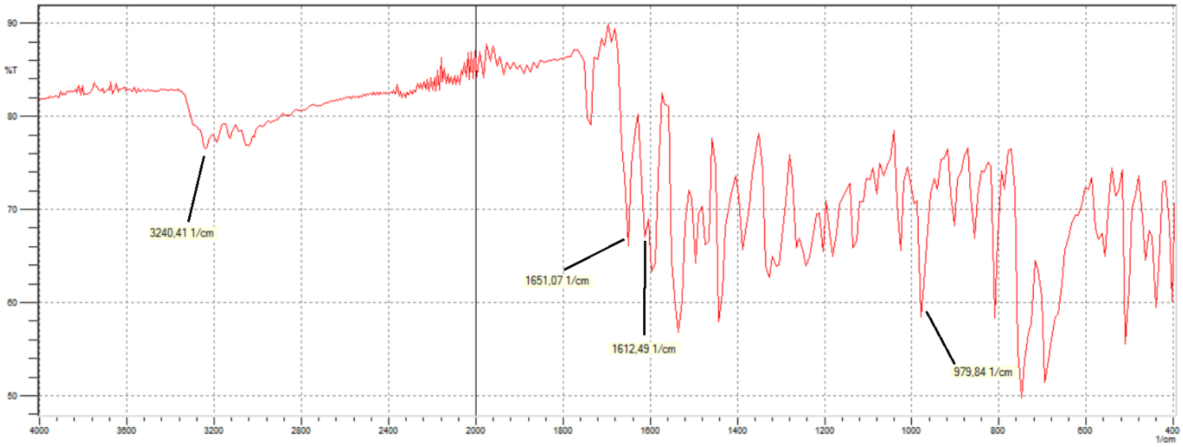

Figure S13. *¹H NMR spectrum of (E)-3-(3,4-Dichlorophenyl)-N-phenylacrylamide* **(LQM328)**

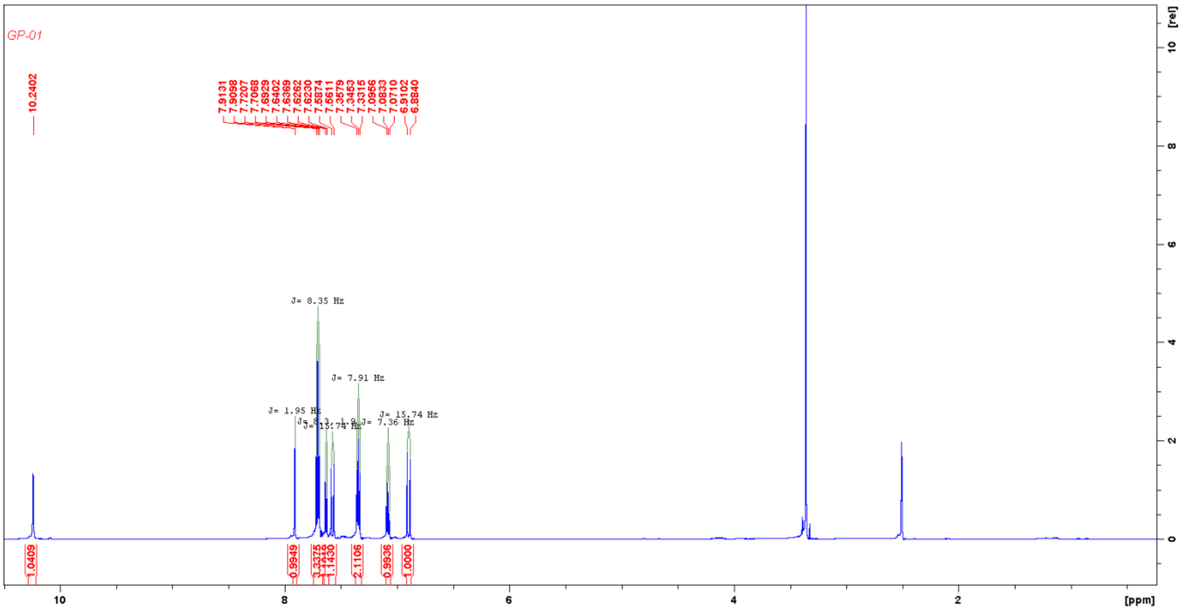

Figure S14. *¹³C NMR spectrum of (E)-3-(3,4-Dichlorophenyl)-N-phenylacrylamide* **(LQM328)**

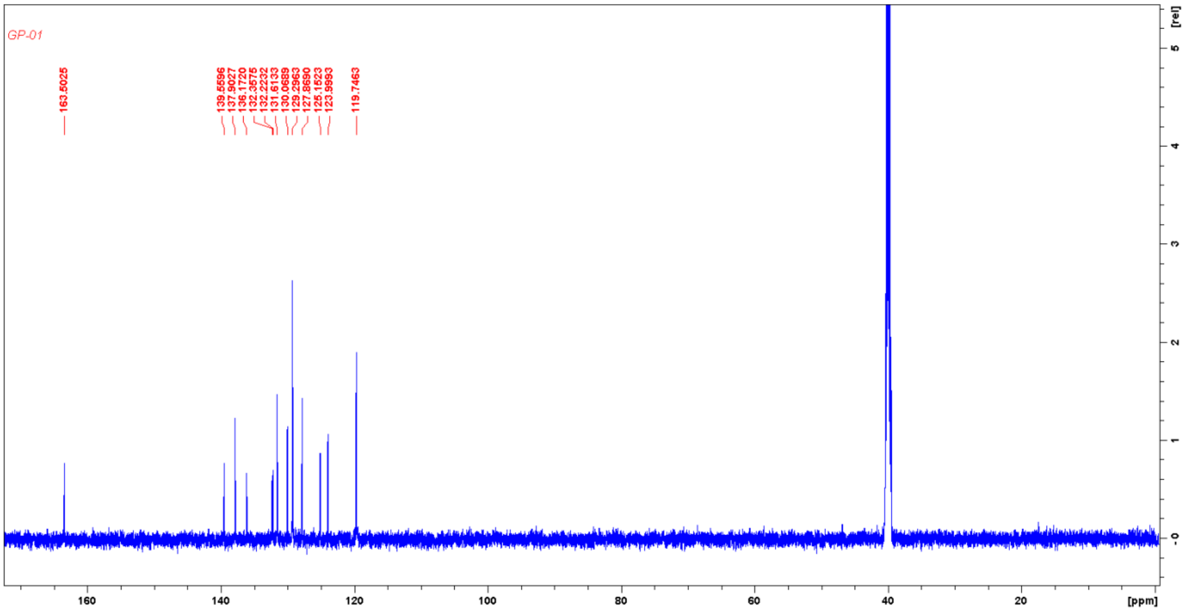

Figure S15. *HPLC chromatogram of (E)-3-([1,1'-Biphenyl]-4-yl)-N-phenylacrylamide* **(LQM329)**

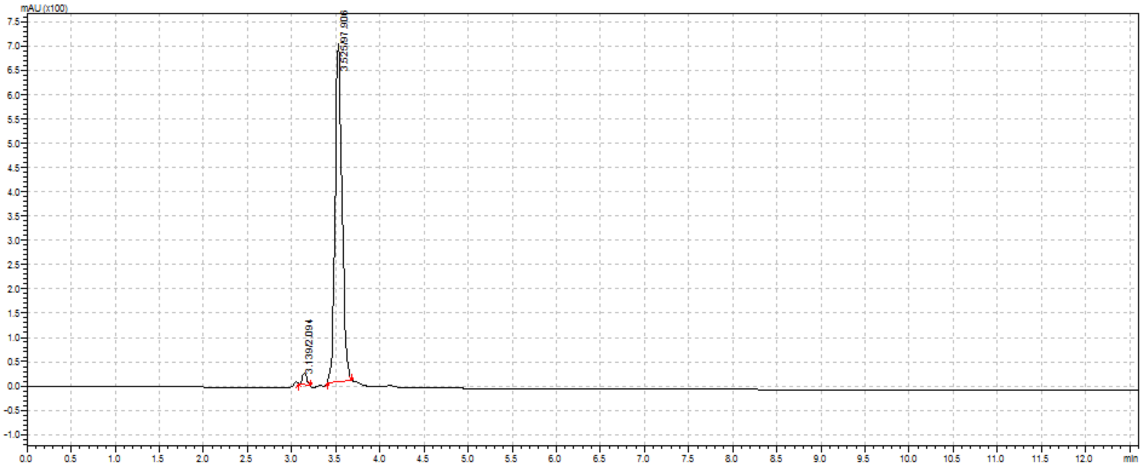

Figure S16. *FT-IR spectrum of (E)-3-([1,1'-Biphenyl]-4-yl)-N-phenylacrylamide* **(LQM329)**

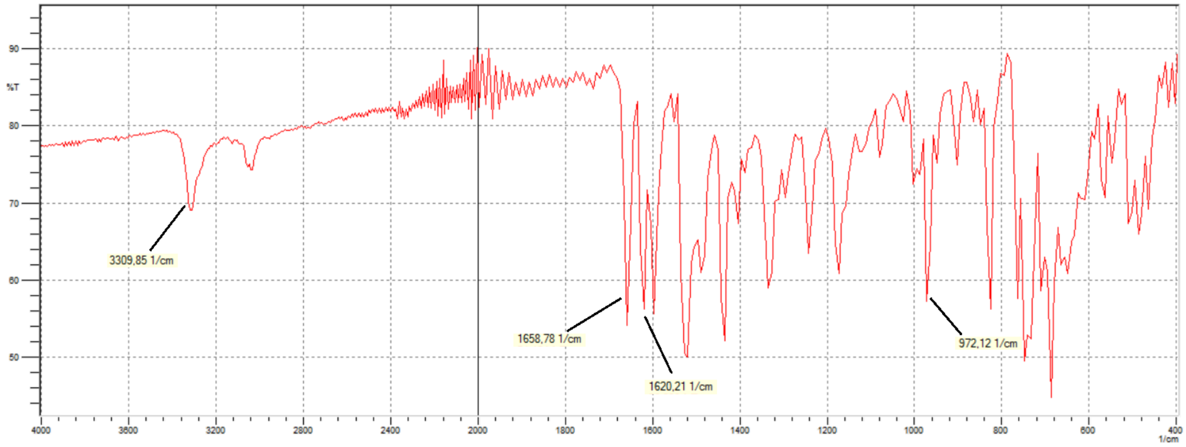

Figure S17. *^{1}H NMR spectrum of (E)-3-([1,1'-Biphenyl]-4-yl)-N-phenylacrylamide* ***(LQM329)***

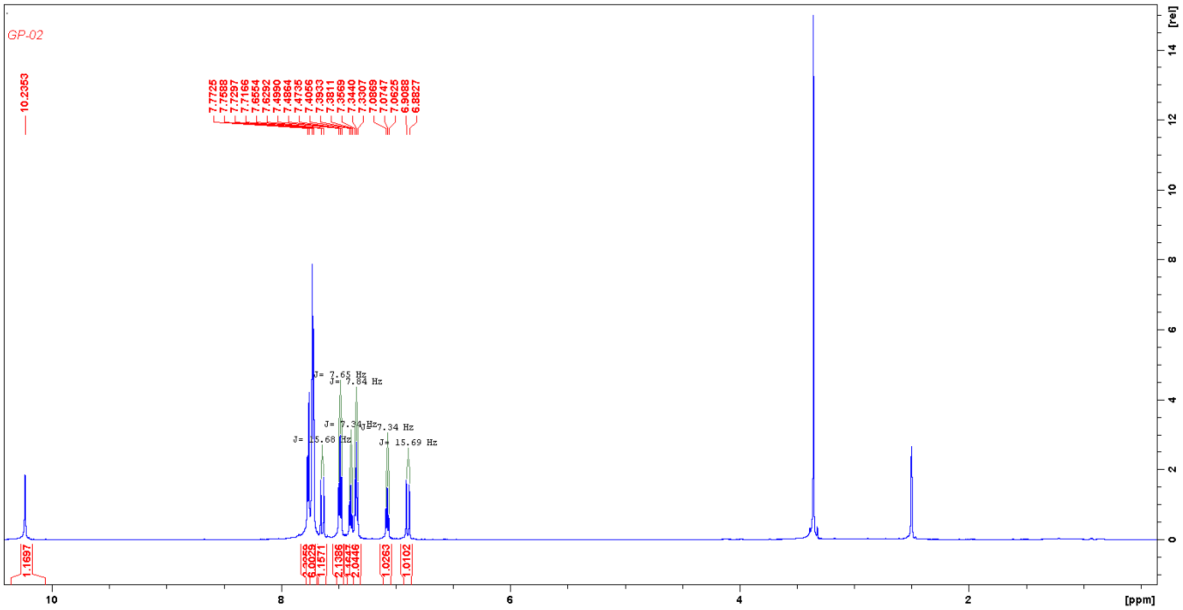

Figure S18. *^{13}C NMR spectrum of (E)-3-([1,1'-Biphenyl]-4-yl)-N-phenylacrylamide* ***(LQM329)***

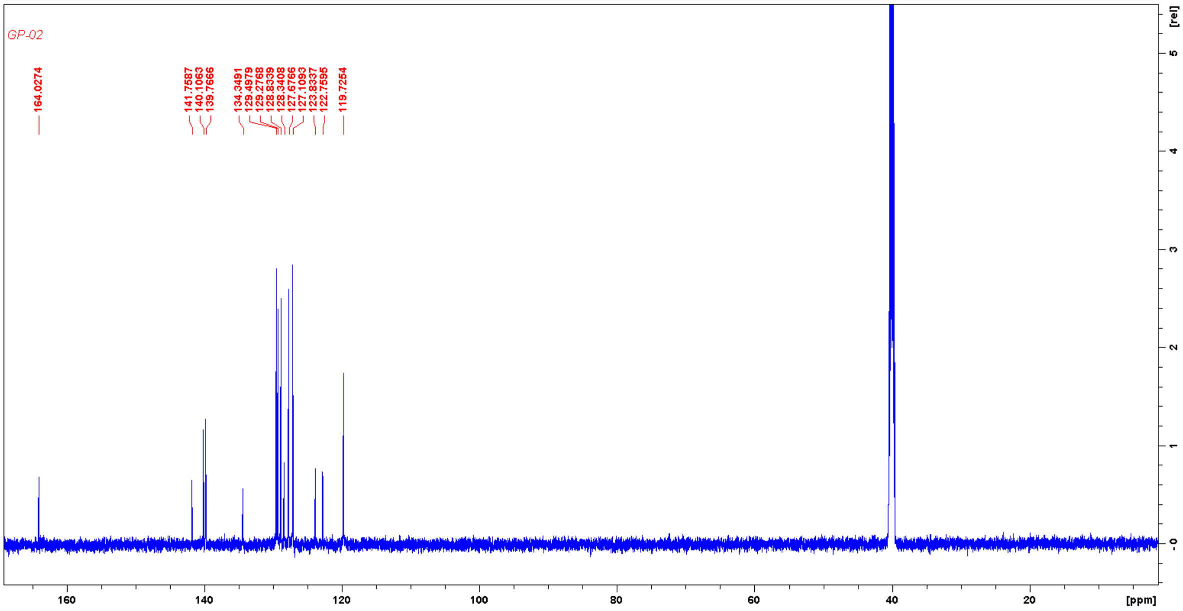

Figure S19. *HPLC chromatogram of (E)-N-Phenyl-3-(4-(trifluoromethyl)phenyl)acrylamide* ***(LQM330)***

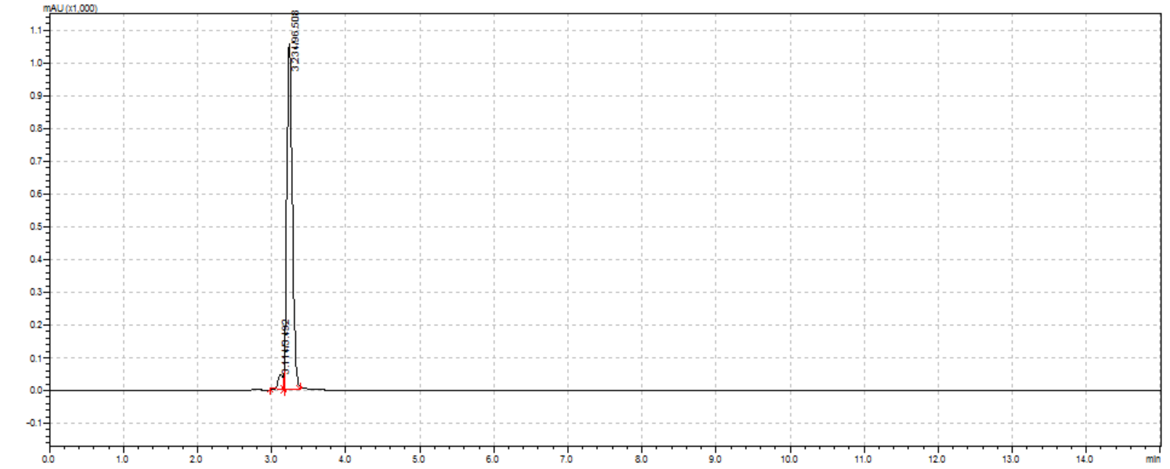

Figure S20. *FT-IR spectrum of (E)-N-Phenyl-3-(4-(trifluoromethyl)phenyl)acrylamide* ***(LQM330)***

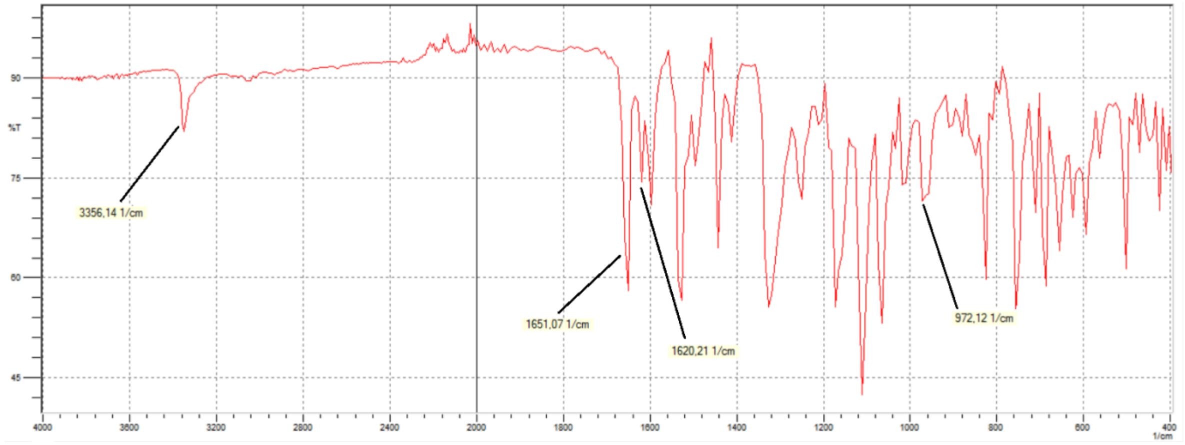

Figure S21. *¹H NMR spectrum of (E)-N-Phenyl-3-(4-(trifluoromethyl)phenyl)acrylamide* ***(LQM330)***

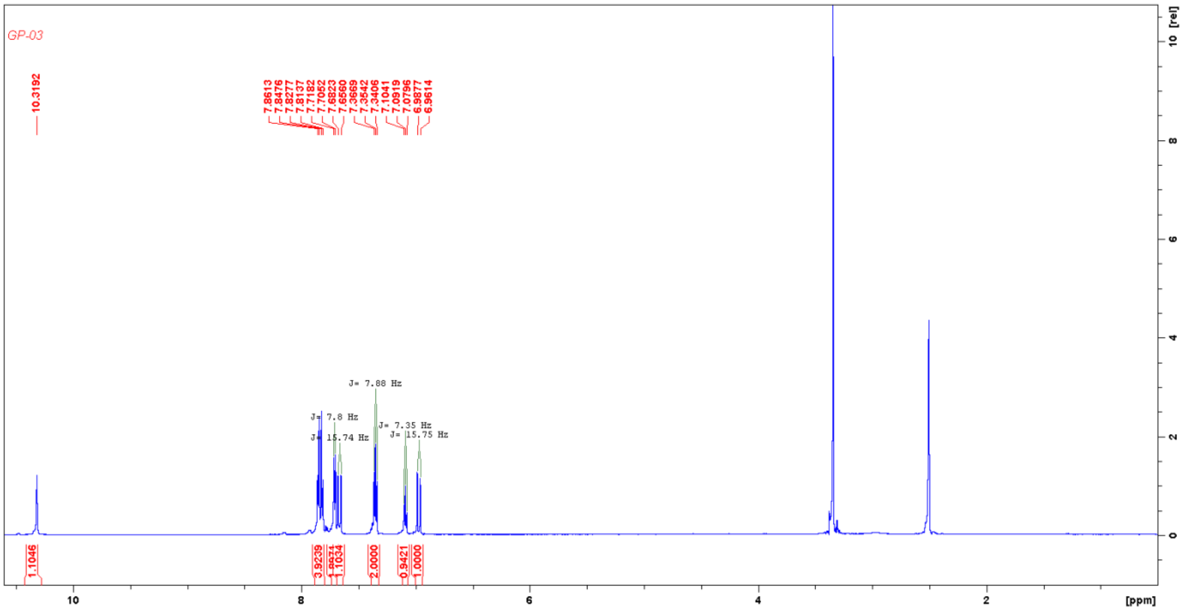

Figure S22. *¹³C NMR spectrum of (E)-N-Phenyl-3-(4-(trifluoromethyl)phenyl)acrylamide* ***(LQM330)***

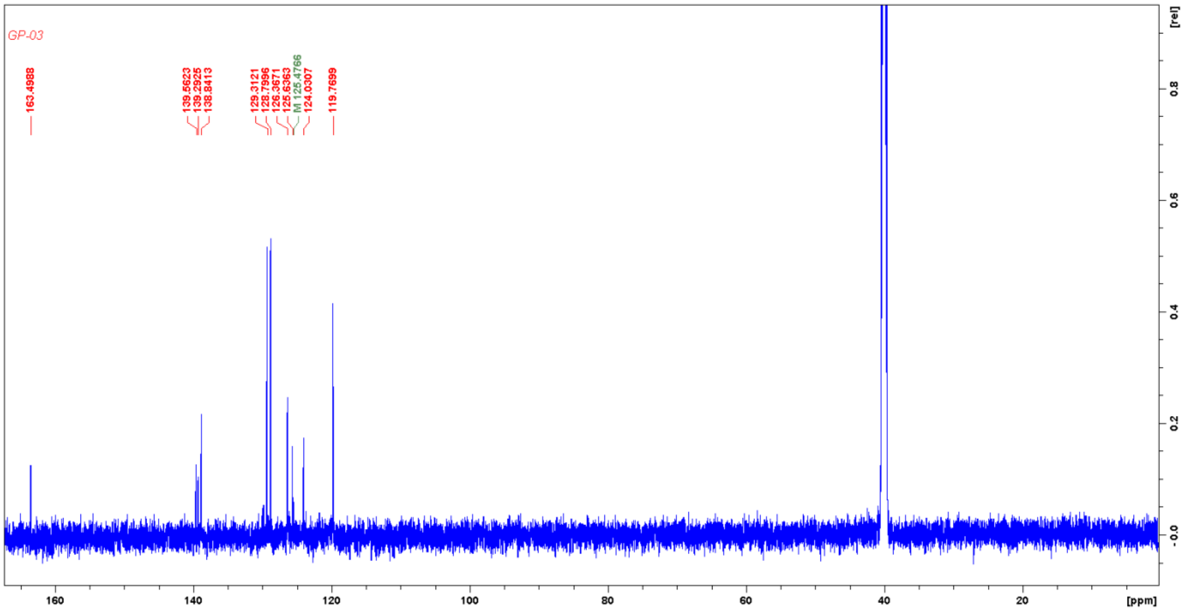

Figure S23. *HPLC chromatogram of (E)-3-(2,3-Dichlorophenyl)-N-phenylacrylamide* ***(LQM331)***

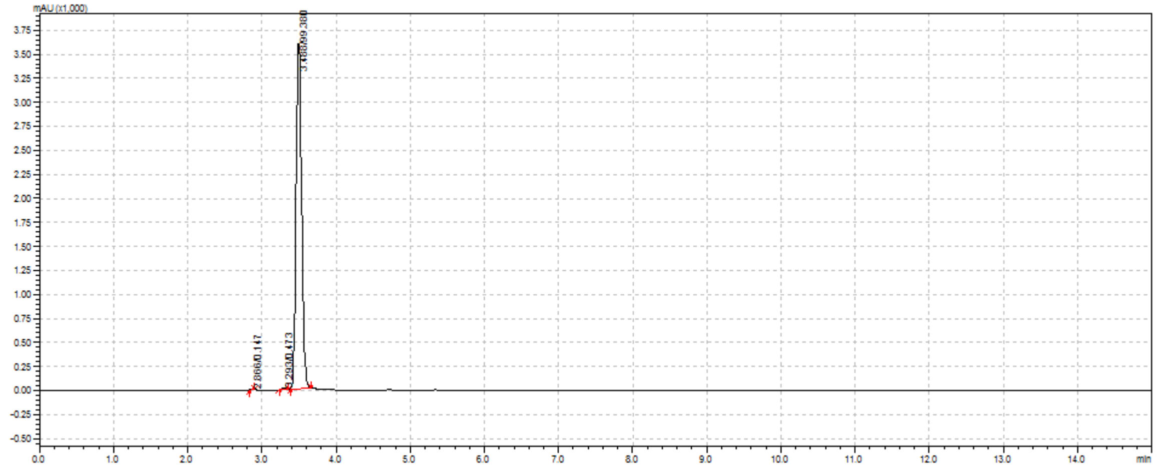

Figure S24. *FT-IR spectrum of (E)-3-(2,3-Dichlorophenyl)-N-phenylacrylamide* ***(LQM331)***

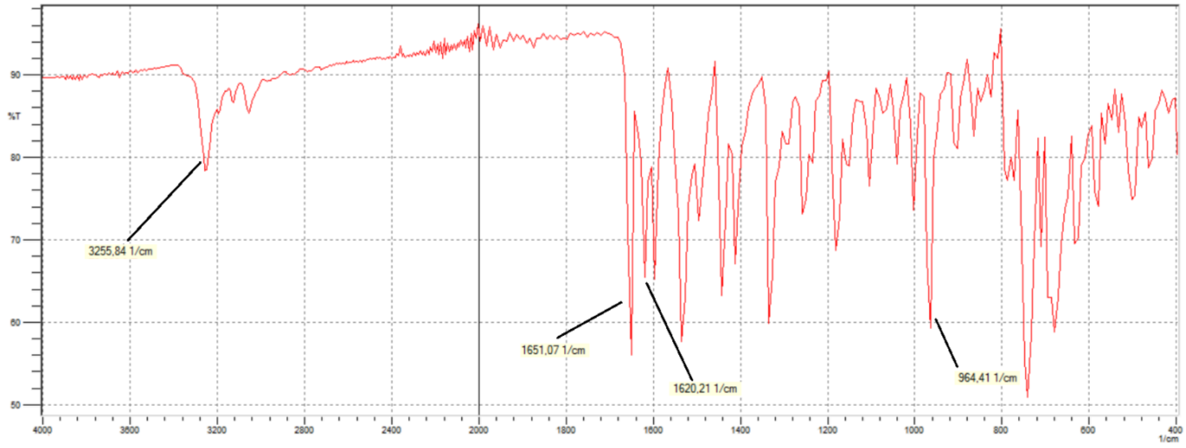

Figure S25. *^{1}H NMR spectrum of (E)-3-(2,3-Dichlorophenyl)-N-phenylacrylamide* ***(LQM331)***

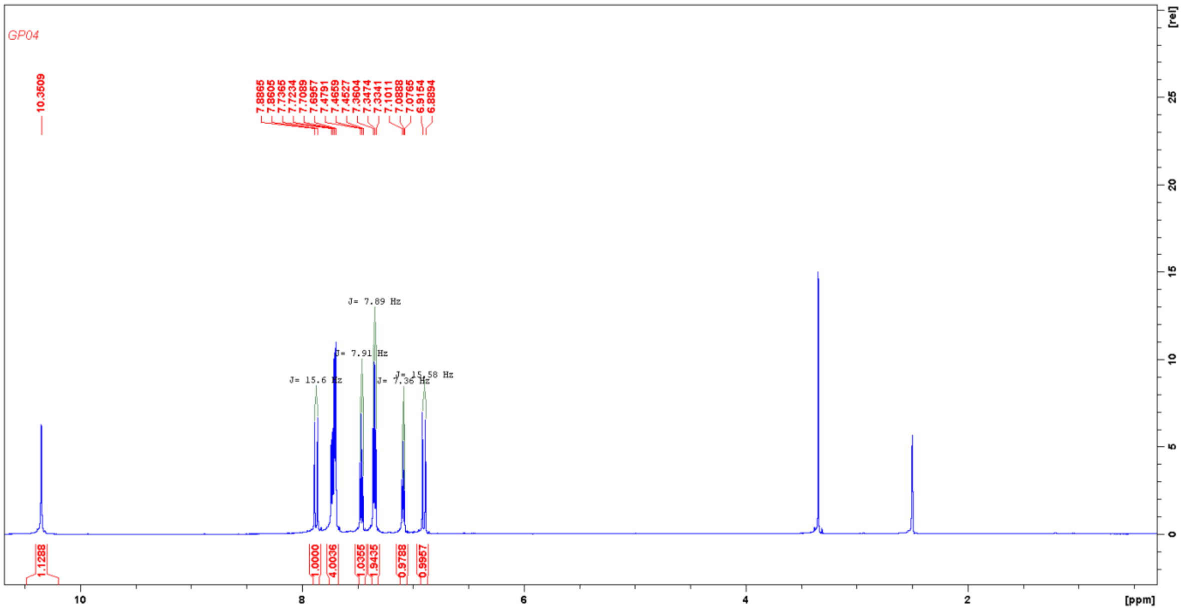

Figure S26. *^{13}C NMR spectrum of (E)-3-(2,3-Dichlorophenyl)-N-phenylacrylamide* ***(LQM331)***

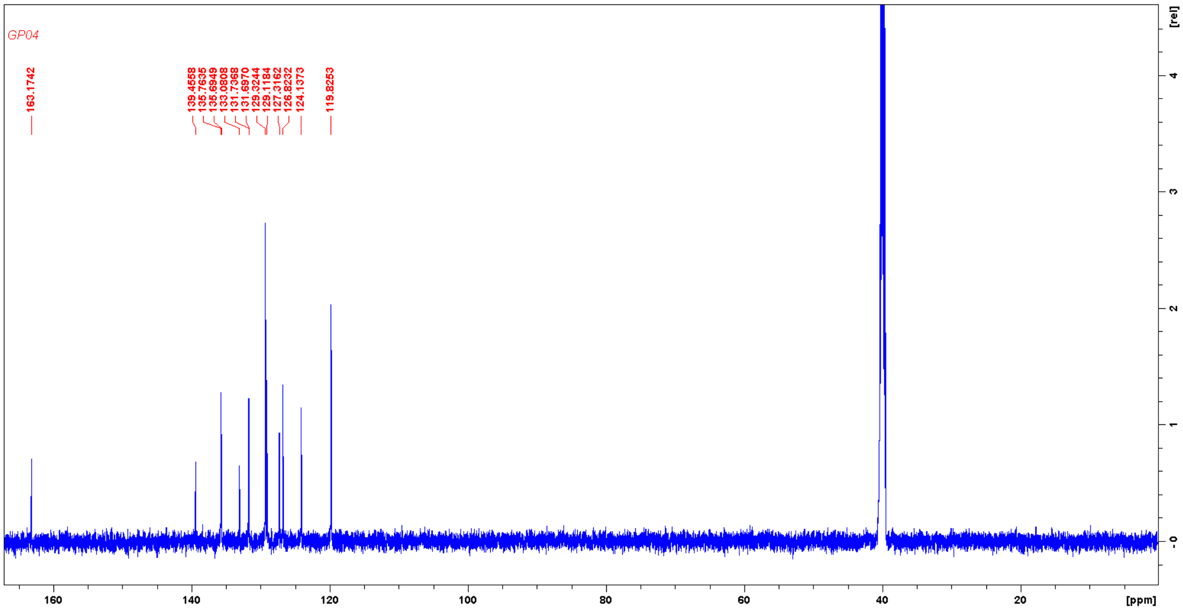

Figure S27. *HPLC chromatogram of (E)-3-(4-Fluorophenyl)-N-phenylacrylamide* ***(LQM332)***

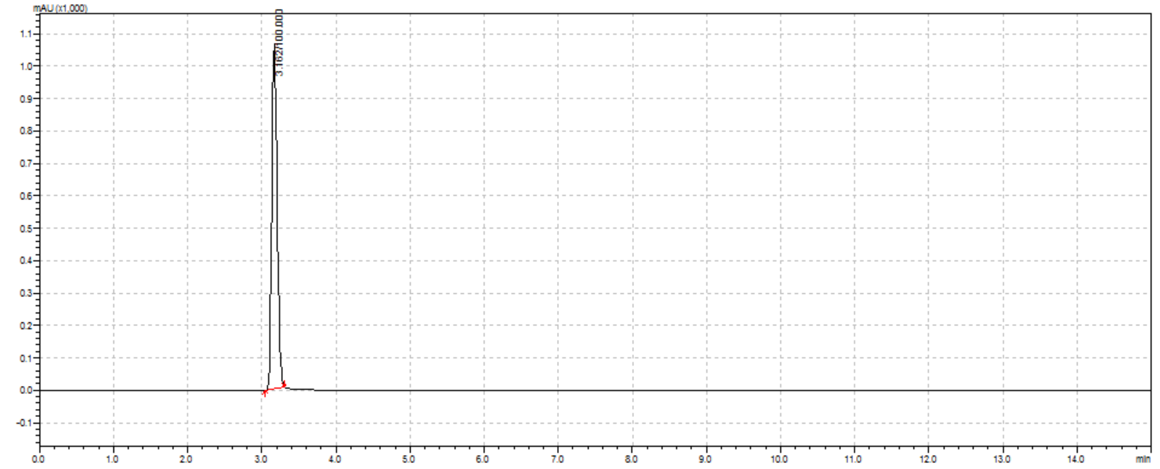

Figure S28. *FT-IR spectrum of (E)-3-(4-Fluorophenyl)-N-phenylacrylamide* ***(LQM332)***

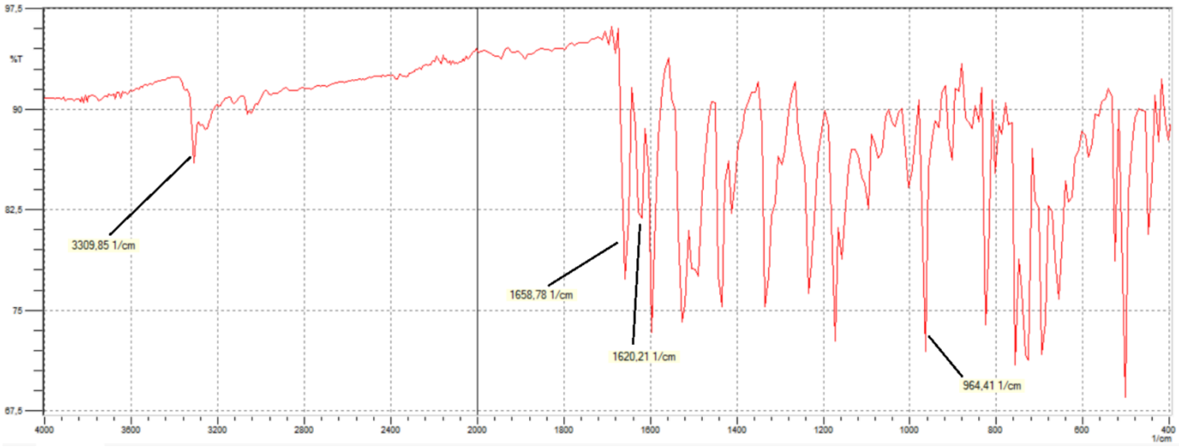

Figure S29. *^{1}H NMR spectrum of (E)-3-(4-Fluorophenyl)-N-phenylacrylamide* ***(LQM332)***

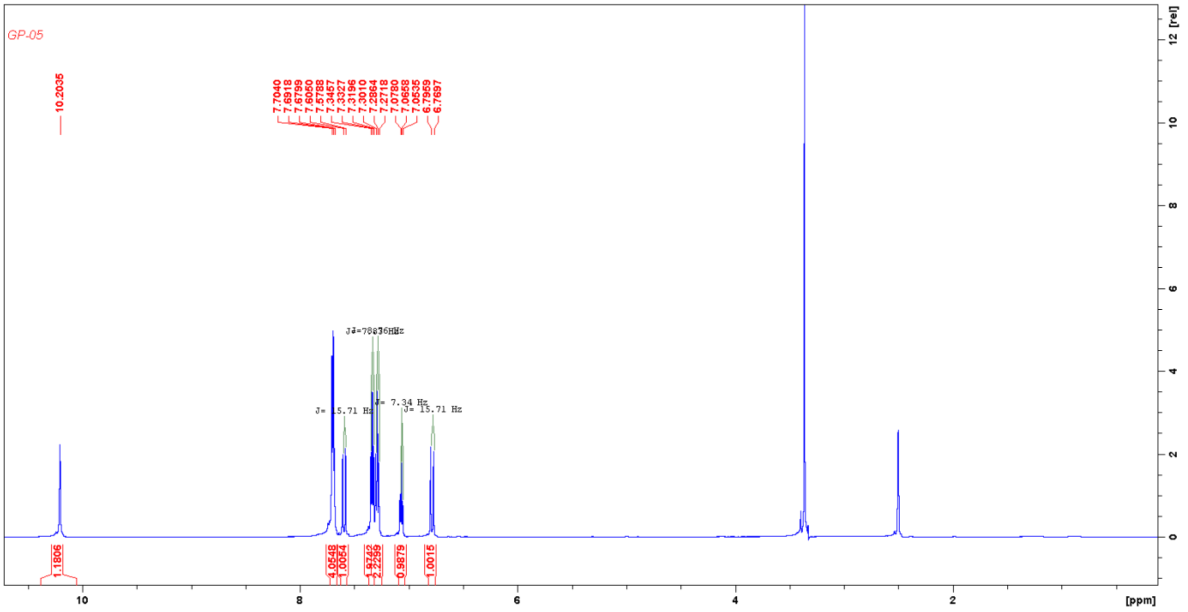

Figure S30. *^{13}C NMR spectrum of (E)-3-(4-Fluorophenyl)-N-phenylacrylamide* ***(LQM332)***

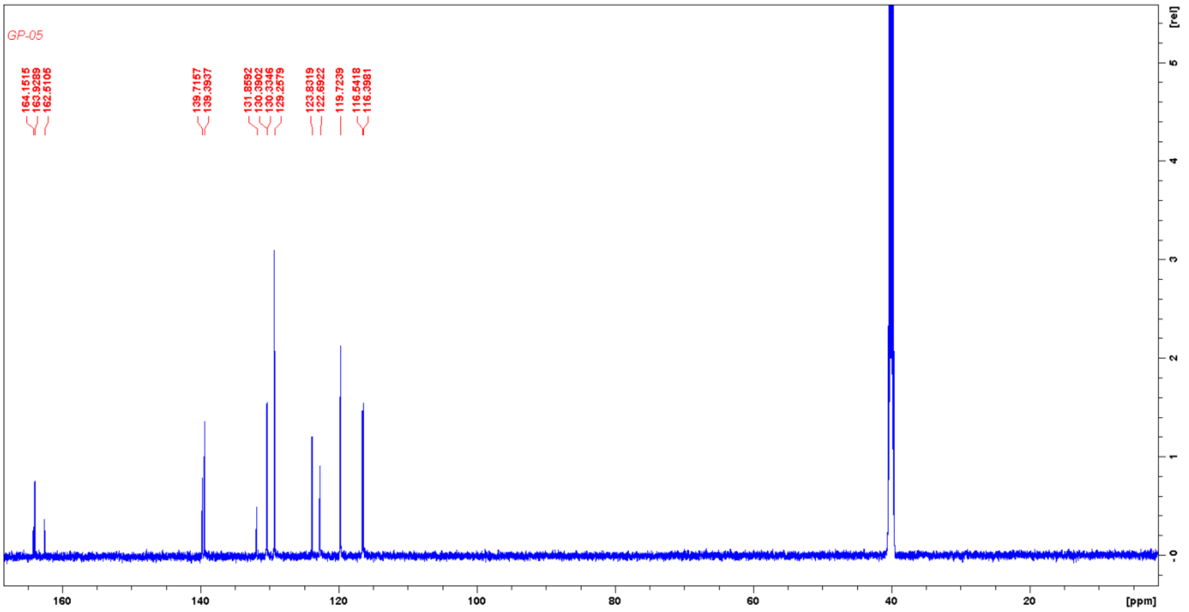

Figure S31. *HPLC chromatogram of (E)-3-(2,4-Dichlorophenyl)-N-phenylacrylamide* ***(LQM333)***

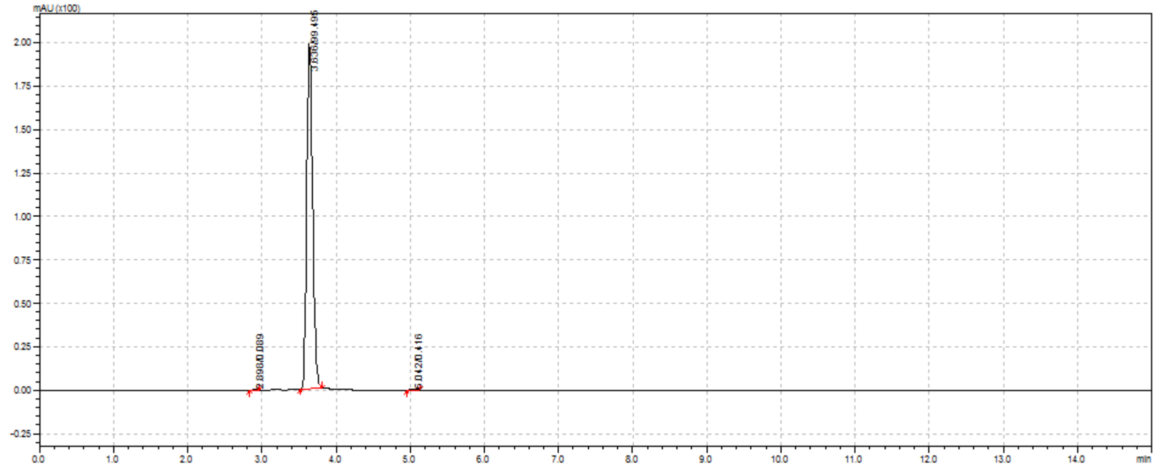

Figure S32. *FT-IR spectrum of (E)-3-(2,4-Dichlorophenyl)-N-phenylacrylamide* ***(LQM333)***

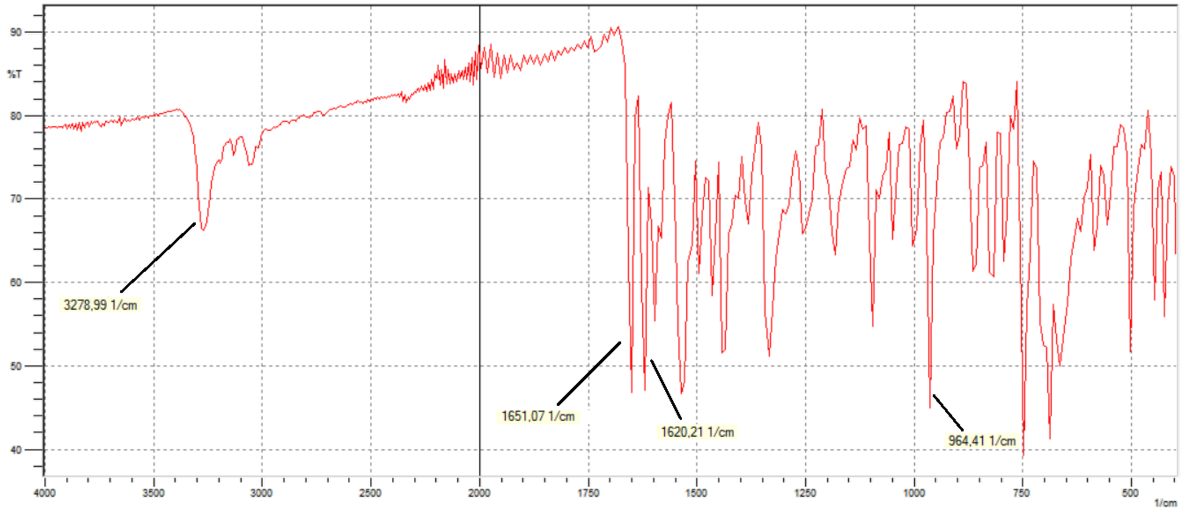

Figure S33. *^{1}H NMR spectrum of (E)-3-(2,4-Dichlorophenyl)-N-phenylacrylamide* ***(LQM333)***

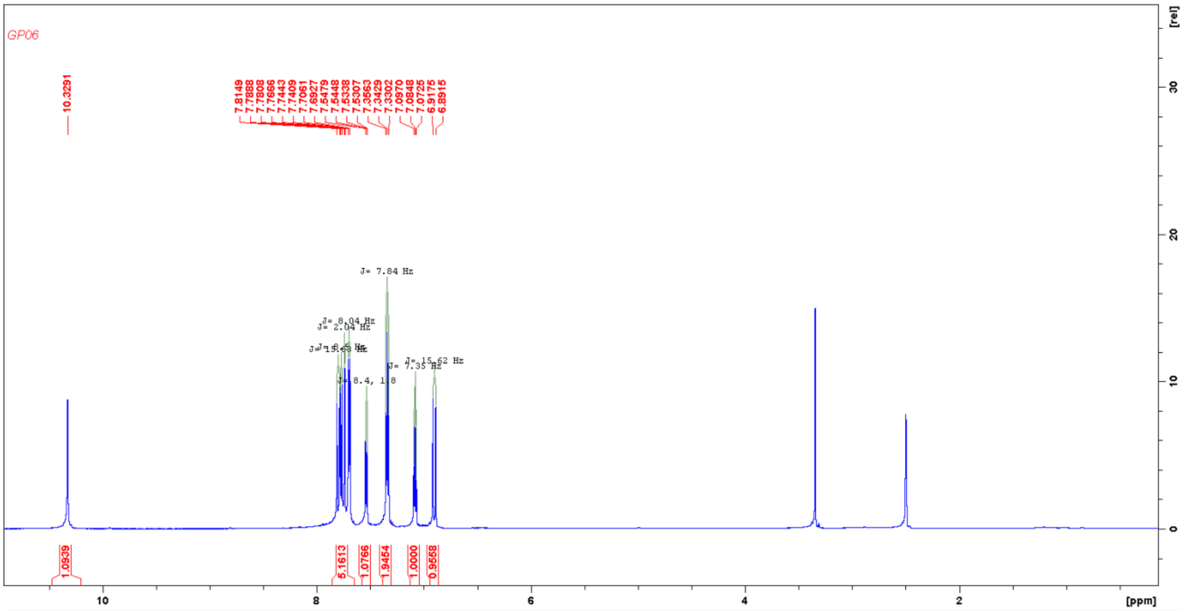

Figure S34. *^{13}C NMR spectrum of (E)-3-(2,4-Dichlorophenyl)-N-phenylacrylamide* ***(LQM333)***

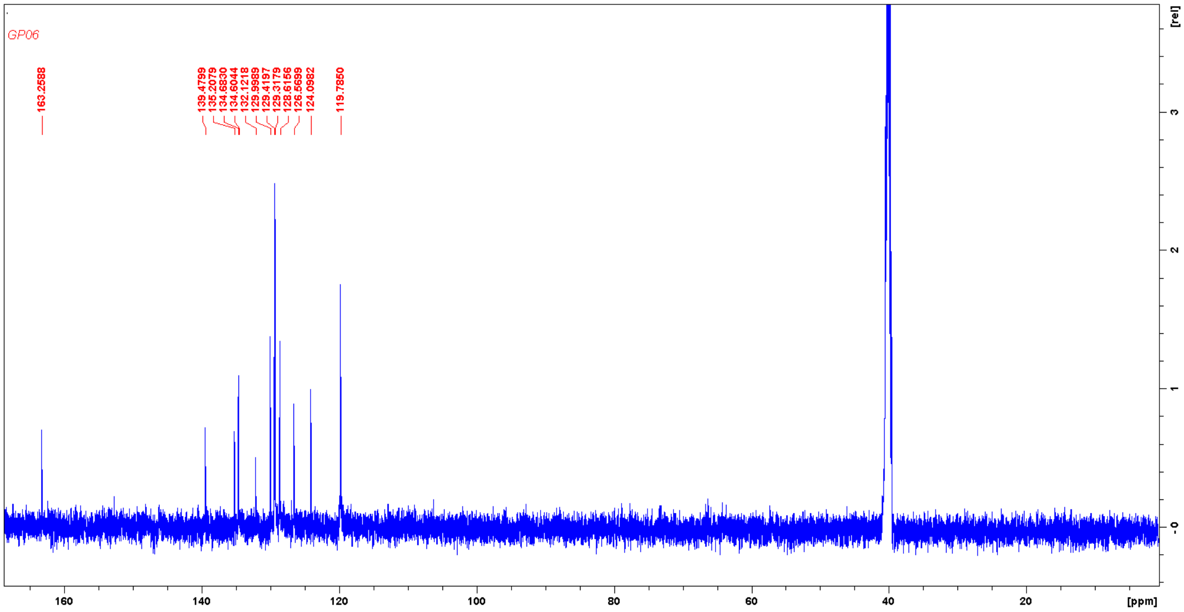

Figure S35. *HPLC chromatogram of (E)-3-(3-Chlorophenyl)-N-phenylacrylamide* ***(LQM334)***

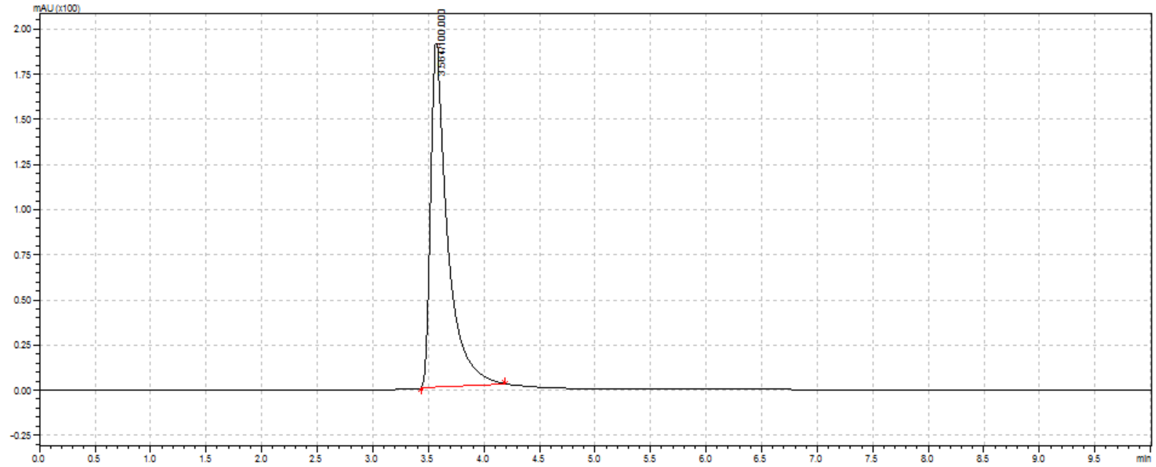

Figure S36. *FT-IR spectrum of (E)-3-(3-Chlorophenyl)-N-phenylacrylamide* ***(LQM334)***

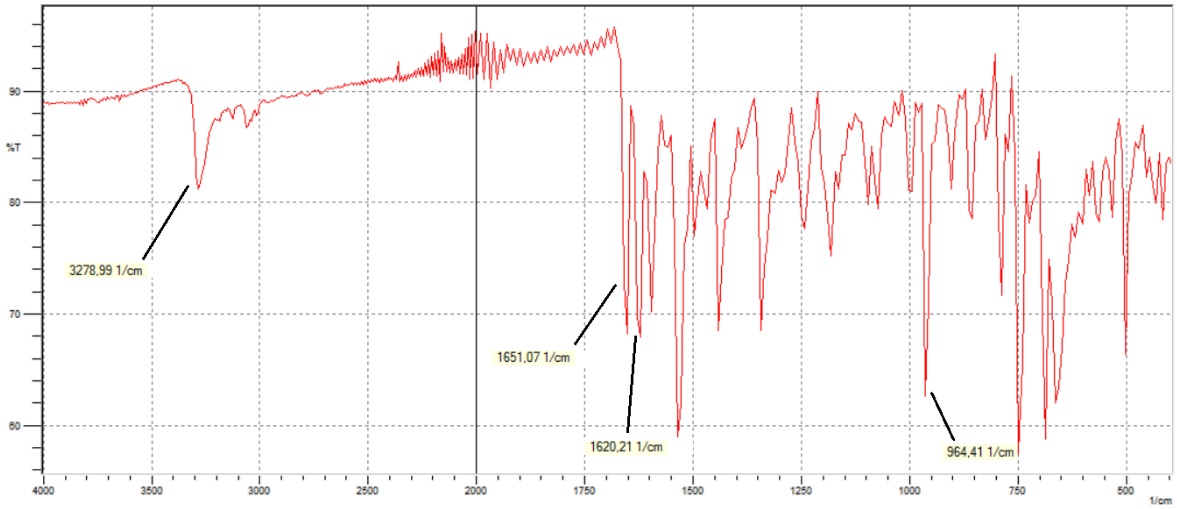

Figure S37. *^{1}H NMR spectrum of (E)-3-(3-Chlorophenyl)-N-phenylacrylamide* ***(LQM334)***

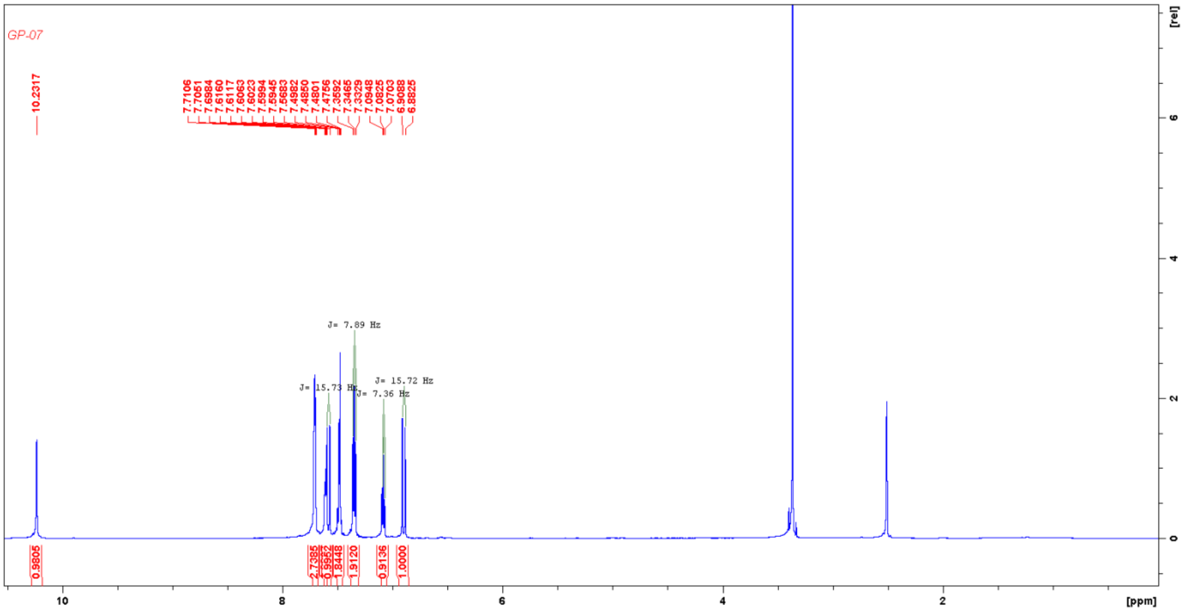

Figure S38. *^{13}C NMR spectrum of (E)-3-(3-Chlorophenyl)-N-phenylacrylamide* ***(LQM334)***

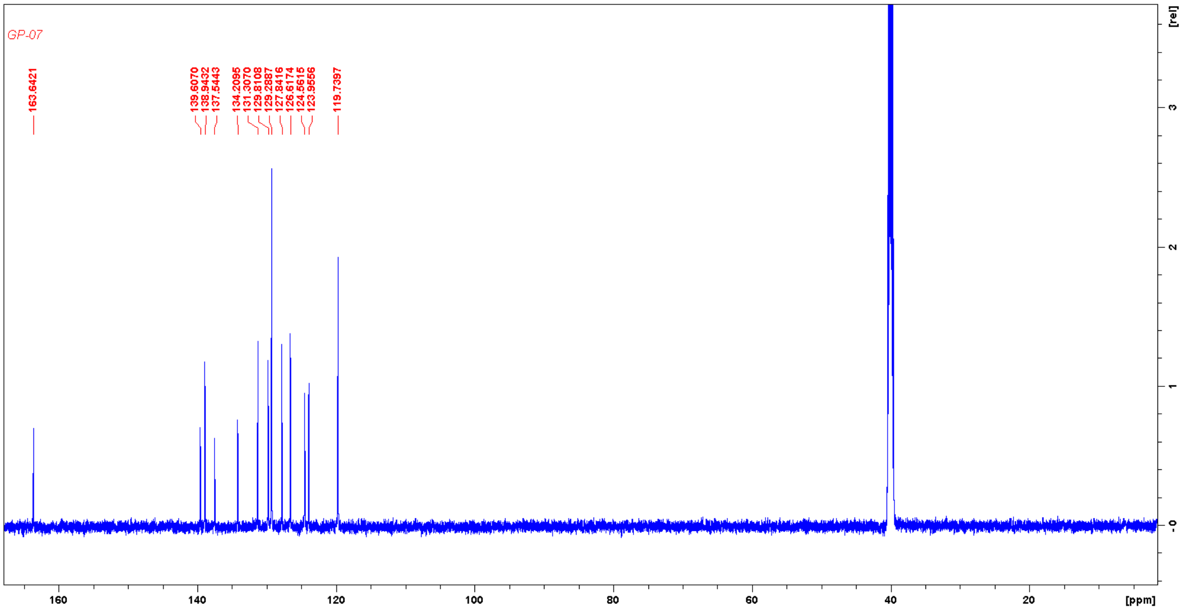

Figure S39. *HPLC chromatogram of (E)-3-(3,4-Dimethoxyphenyl)-N-phenylacrylamide* ***(LQM335)***

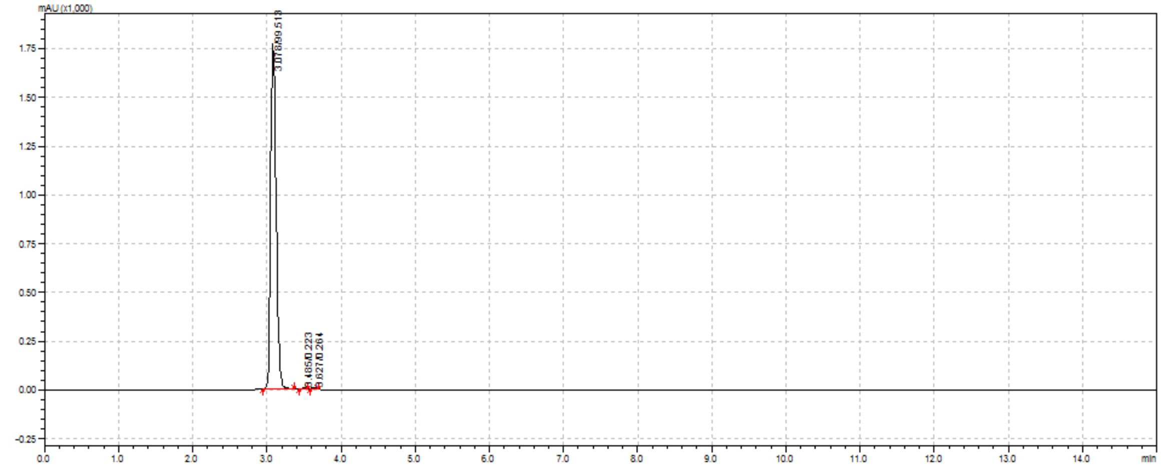

Figure S40. *FT-IR spectrum of (E)-3-(3,4-Dimethoxyphenyl)-N-phenylacrylamide* ***(LQM335)***

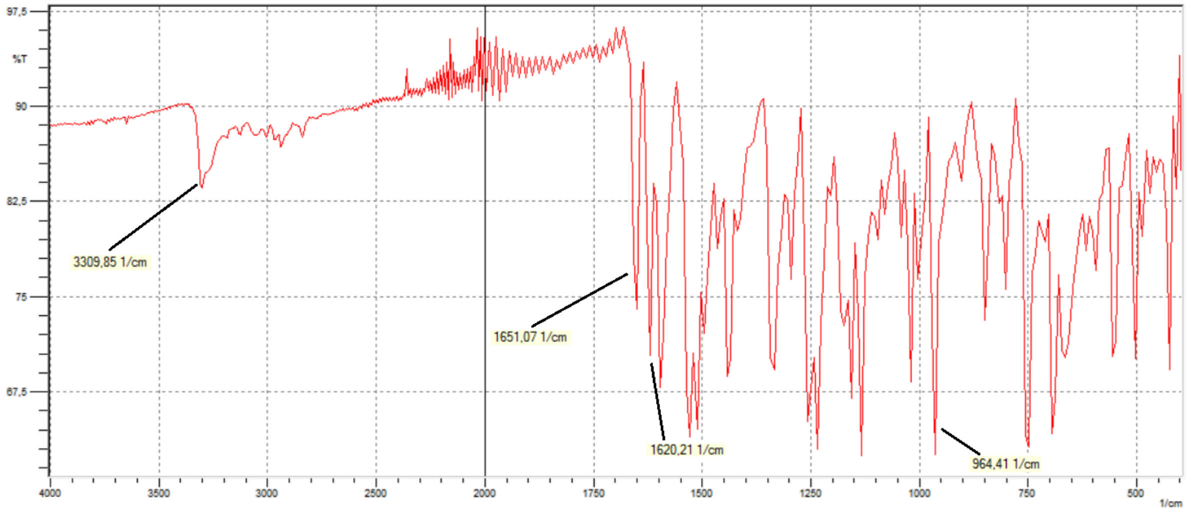

Figure S41. *^{1}H NMR spectrum of (E)-3-(3,4-Dimethoxyphenyl)-N-phenylacrylamide* **(LQM335)**

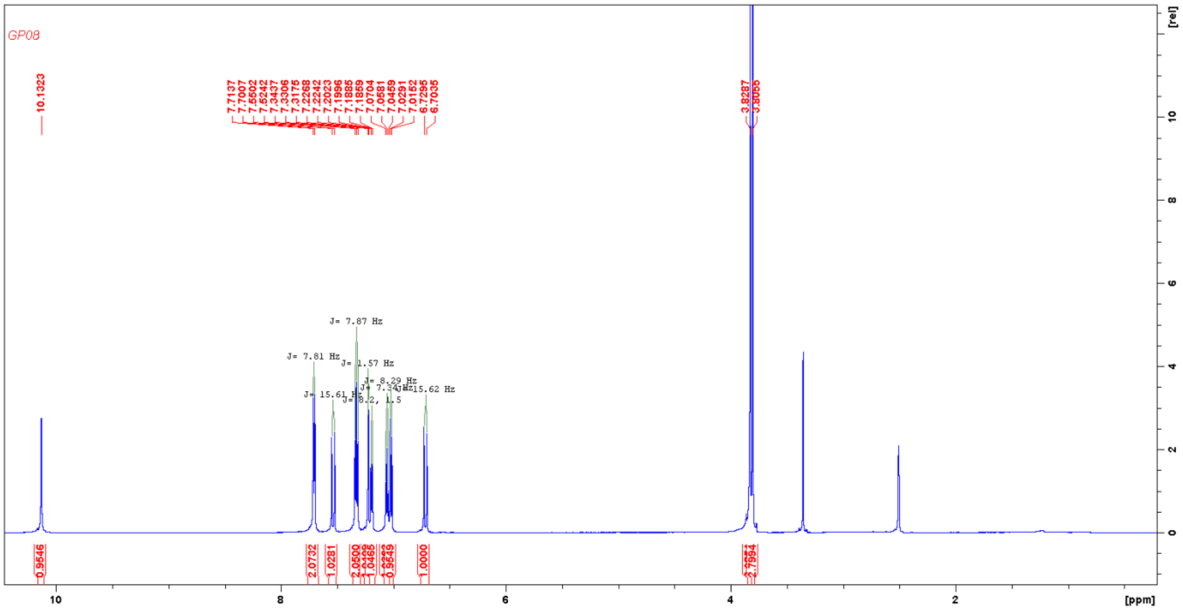

Figure S42. *^{13}C NMR spectrum of (E)-3-(3,4-Dimethoxyphenyl)-N-phenylacrylamide* **(LQM335)**

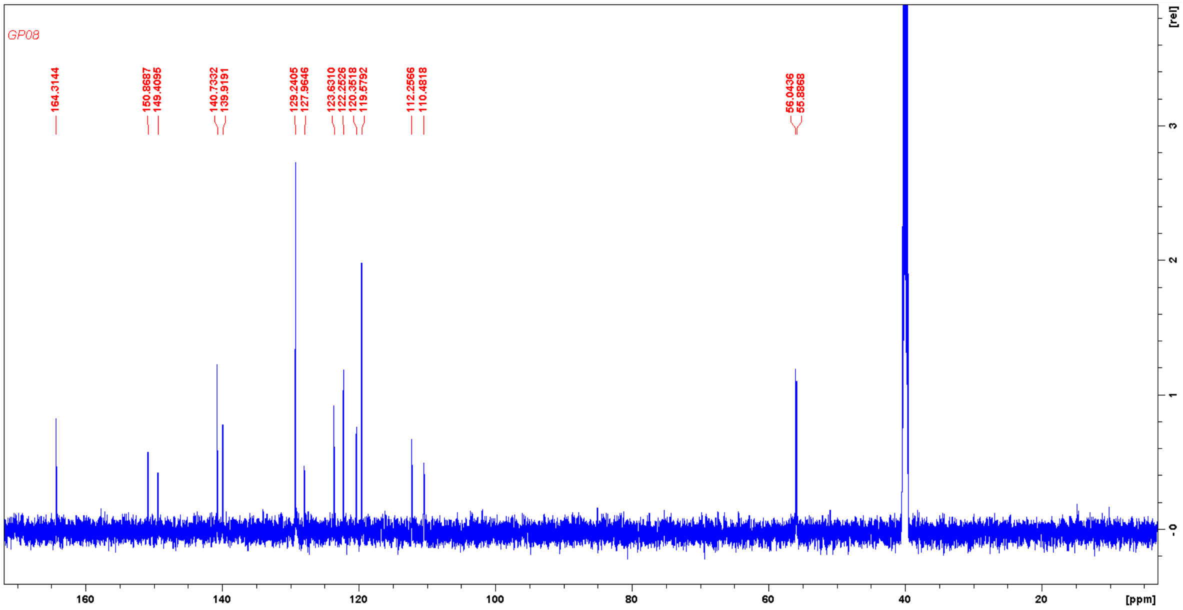

Figure S43. *HPLC chromatogram of (E)-3-(2-Methoxyphenyl)-N-phenylacrylamide* ***(LQM336)***

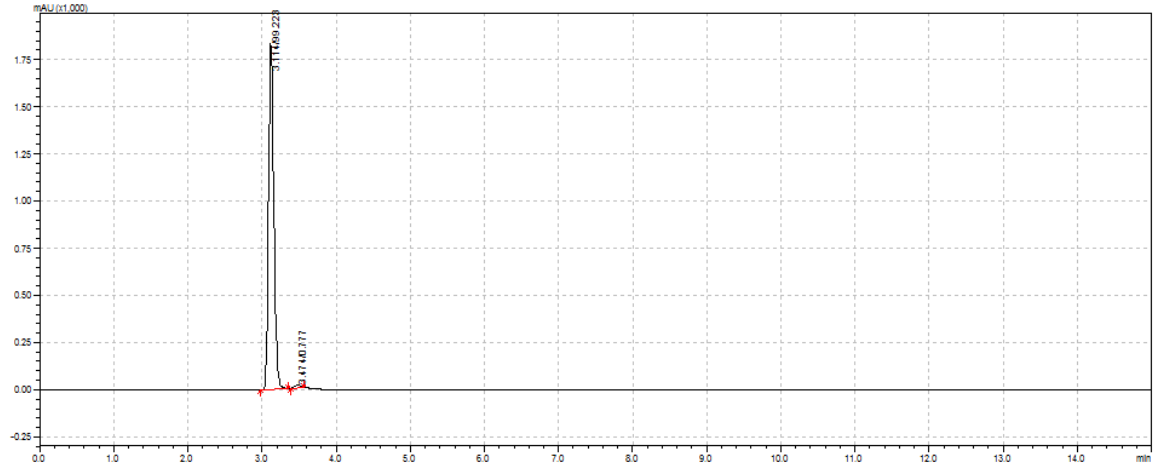

Figure S44. *FT-IR spectrum of (E)-3-(2-Methoxyphenyl)-N-phenylacrylamide* ***(LQM336)***

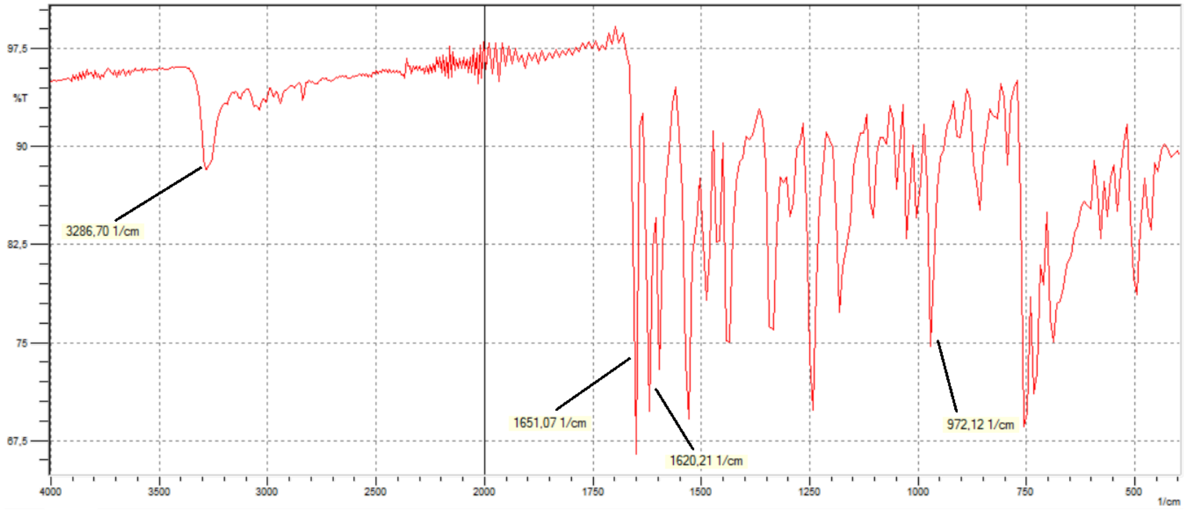

Figure S45. *^{1}H NMR spectrum of (E)-3-(2-Methoxyphenyl)-N-phenylacrylamide* ***(LQM336)***

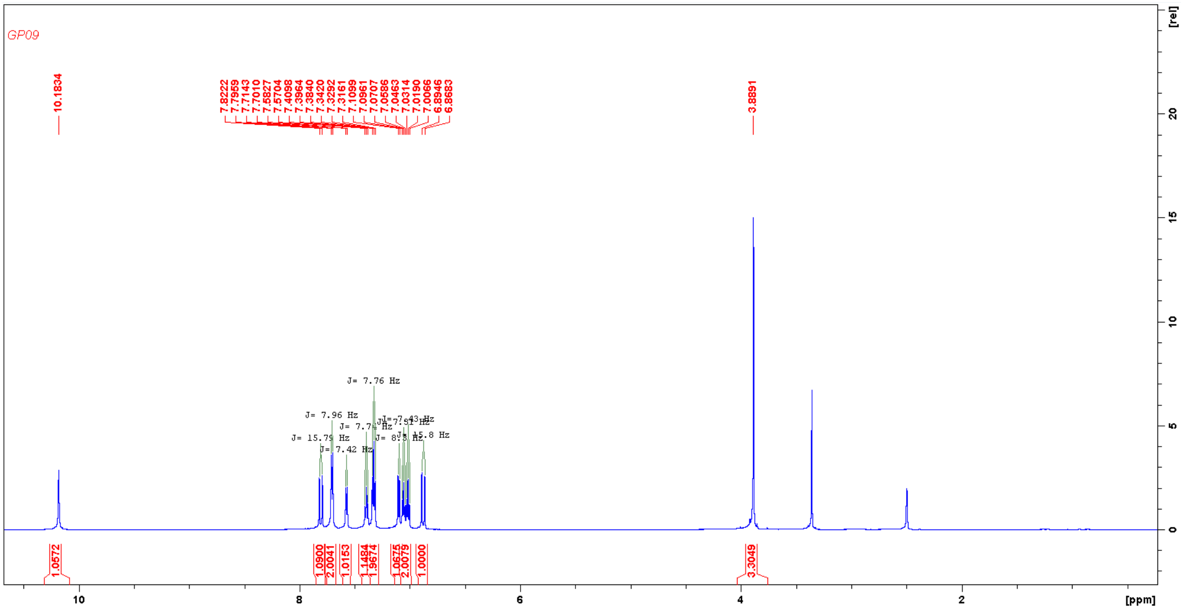

Figure S46. *^{13}C NMR spectrum of (E)-3-(2-Methoxyphenyl)-N-phenylacrylamide* ***(LQM336)***

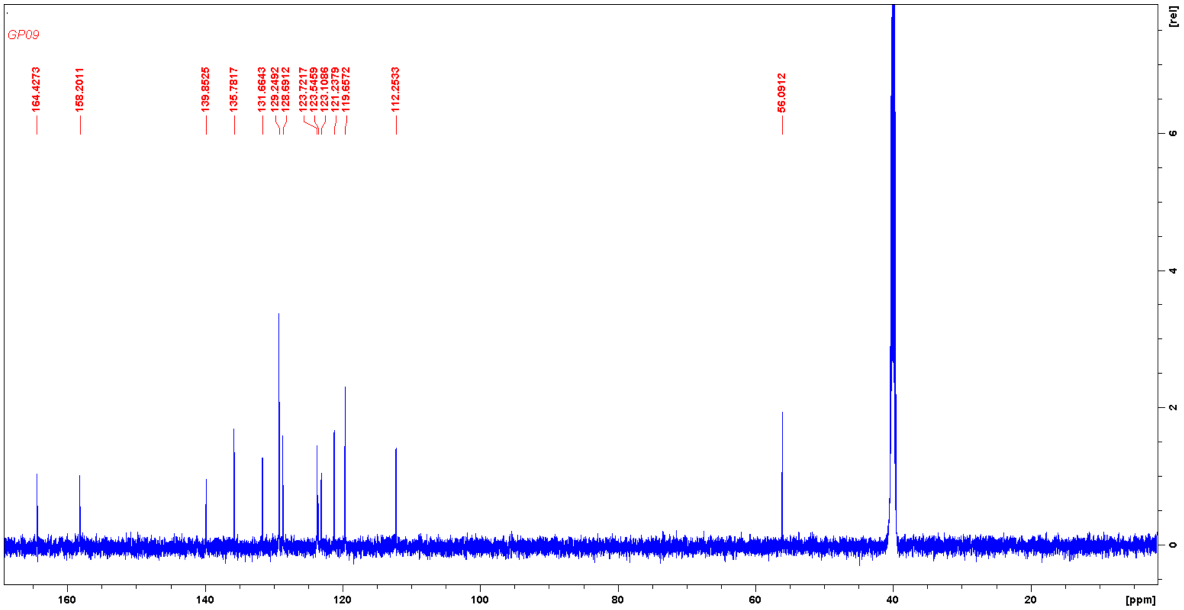

Figure S47. *HPLC chromatogram of (E)-3-([1,1'-Biphenyl]-2-yl)-N-phenylacrylamide* ***(LQM337)***

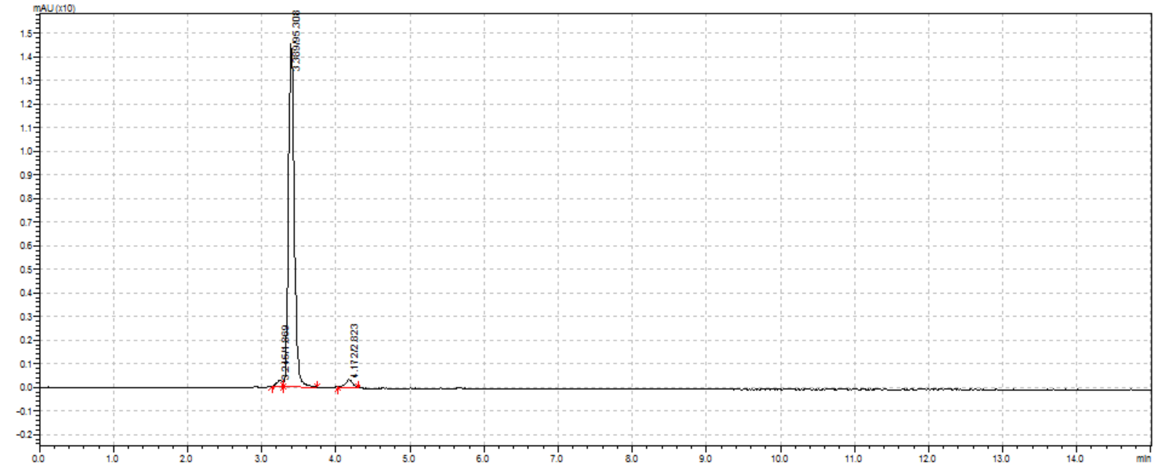

Figure S48. *FT-IR spectrum of (E)-3-([1,1'-Biphenyl]-2-yl)-N-phenylacrylamide* ***(LQM337)***

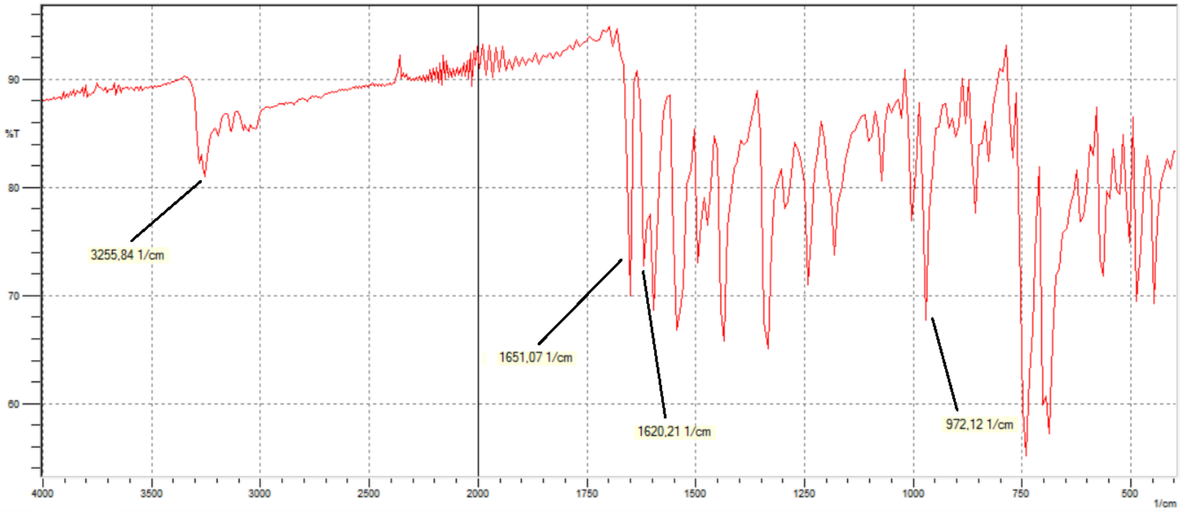

Figure S49. *^{1}H NMR spectrum of (E)-3-([1,1'-Biphenyl]-2-yl)-N-phenylacrylamide* ***(LQM337)***

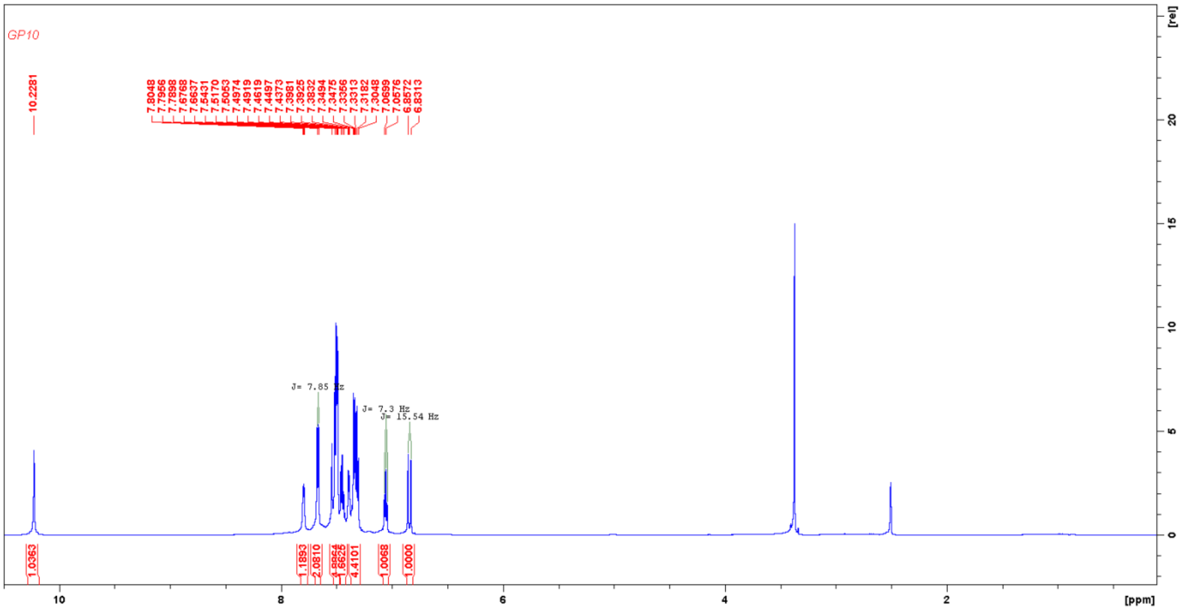

Figure S50. *^{13}C NMR spectrum of (E)-3-([1,1'-Biphenyl]-2-yl)-N-phenylacrylamide* ***(LQM337)***

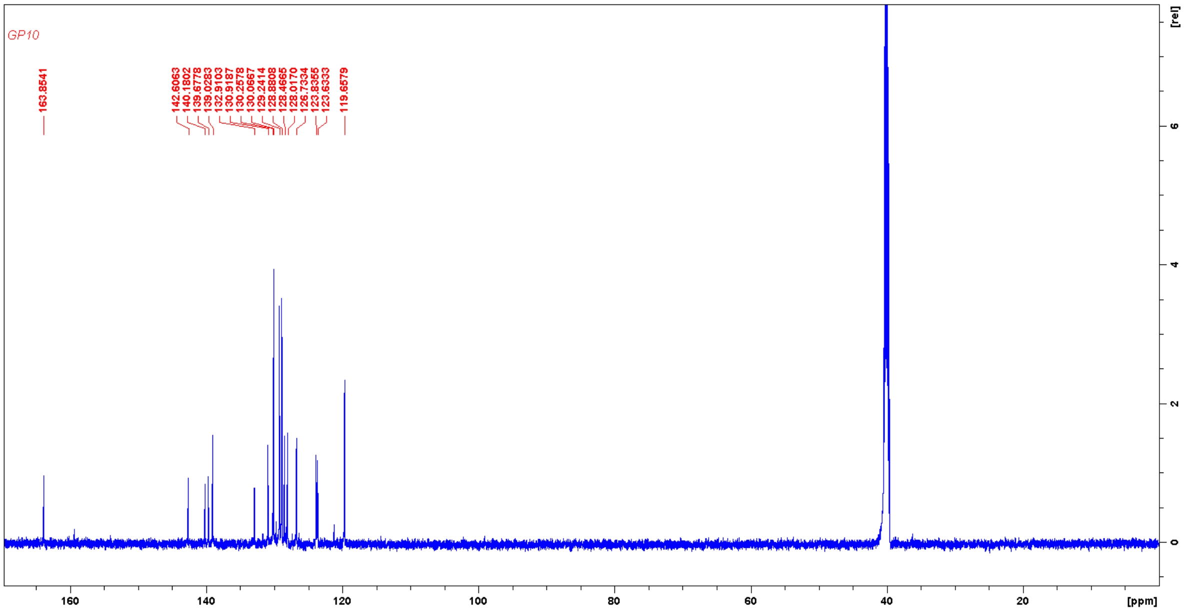

Supplement: Supplementary file 1 [file pharmaceuticals-13-00141-s001.pdf]
